# Supplementary material for: Efficient generation of germline chimeras in a non-rodent species using rabbit induced pluripotent stem cells
Source: Nat Commun. 2025 Jun 4;16:5165. doi: 10.1038/s41467-025-60314-2 (PMC12134177; doi:10.1038/s41467-025-60314-2)

Supplementary information

Supplementary Figure 1
Supplementary Figure 2
Supplementary Figure 3
Supplementary Figure 4
Supplementary Figure 5
Supplementary Figure 6
Supplementary Figure 7
Supplementary Figure 8
Supplementary Figure 9
Supplementary Figure 10
Supplementary Figure 11
Supplementary Figure 12
Supplementary Figure 13
Supplementary Figure 14
Supplementary Figure 15
Supplementary Table 1
Supplementary Table 2
Supplementary Table 3
Supplementary Table 4
Uncropped Western blots

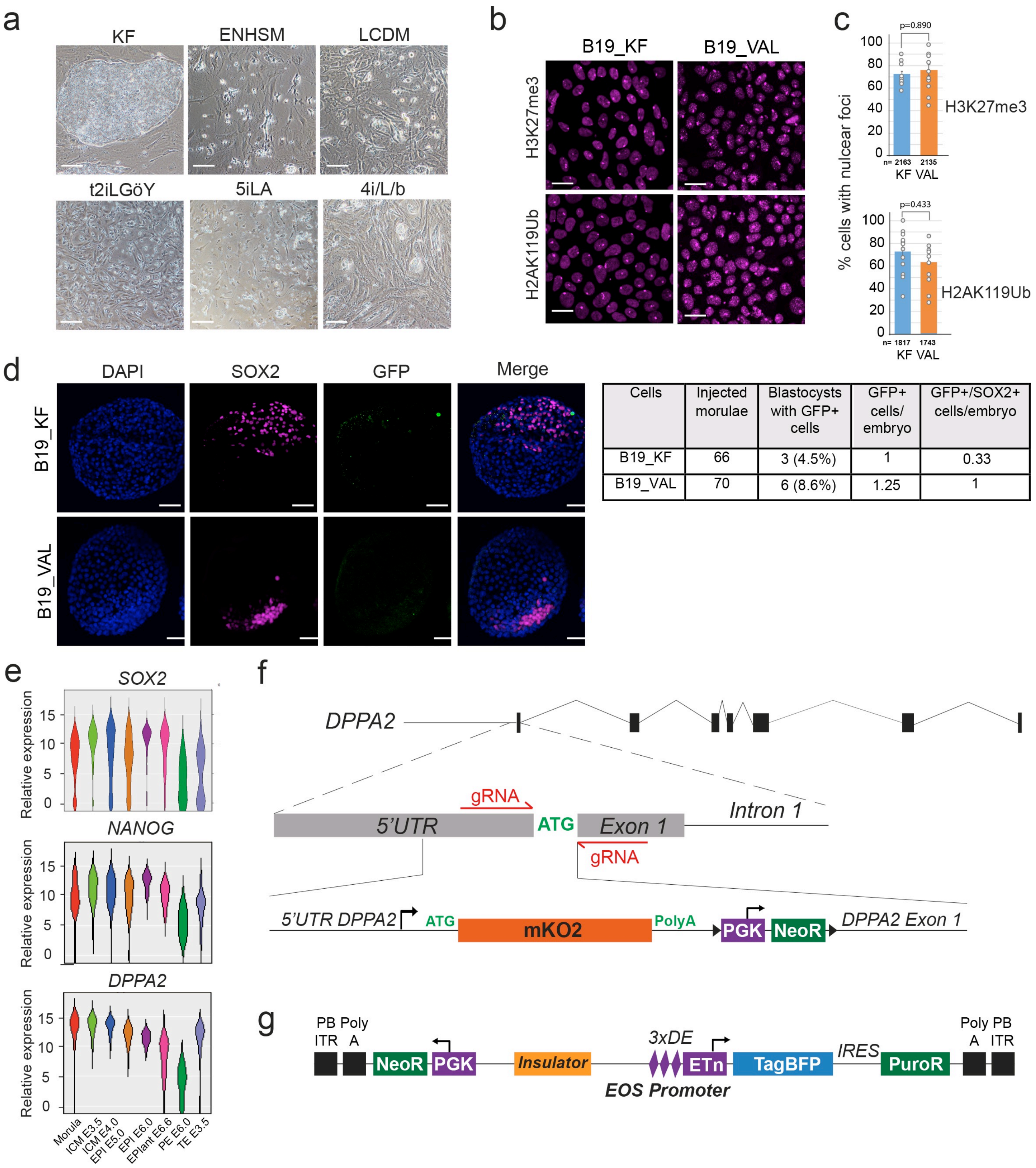

Cells	Injected morulae	Blastocysts with GFP+ cells	GFP+ cells/ embryo	GFP+/SOX2+ cells/embryo
B19_KF	66	3 (4.5%)	1	0.33
B19_VAL	70	6 (8.6%)	1.25	1

Supplementary Figure 1: Design of a novel culture regimen and reporter cell line for naïve pluripotency. (**a**) Phase contrast pictures of control B19 cells grown in KOSR+FGF (KF), and the same cells after two weeks in various naïve media (HENSM, LCDM, t2iLGöY, 5iLA, and 4i/L/b). Scale bars, 100µm. (**b**) Immunostaining of H3K27me3 and H2AK119Ub histone marks in B19_KF and B19_VAL cells (n = 4 independent experiments). Scale bars, 30µm. (**c**) Histograms showing the percentage of cells with H3K27me3 and H2AK119Ub nuclear foci in B19_KF and B19_VAL_48h cultures. Values represent means ± SD calculated from a minimum of 1,700 cells per condition (exact n indicated), across three independent replicates. Each dot represents one replicate; bars indicate mean ± SEM. Comparisons between KF and VAL conditions were performed using a two-sided Welch's t-test (p-values indicated above each chart). (**d**) Immunostaining for GFP and SOX2 in late-blastocyst-stage rabbit embryos (E5.0, 3DIV) following microinjection of B19_KF and B19_VAL cells into morula-stage (E2.8) embryos. Scale bars, 50µm. Table summarizes, for each cell line, the number of injected morulae, blastocysts containing GFP^+ cells, average number of GFP^+ cells per embryo, and $GFP^+/SOX2^+$ cells per embryo. (**e**) Violin plots of SOX2, NANOG, and DPPA2 expression based on single-cell RNA sequencing data [from Bouchereau *et al.*, 2022]; E, embryonic day; ICM, inner cell mass;

EPI, epiblast; EPIant, anterior epiblast; PE, primitive endoderm; TE, trophectoderm. (**f**) Schematic of the CRISPR/Cas9-mediated strategy for inserting mKO2 into the DPPA2 locus of GFP-expressing B19_KF cells. (**g**) Diagram of the EOS-TagBFP PiggyBac expression vector used to generate NaiveRep_KF cells.

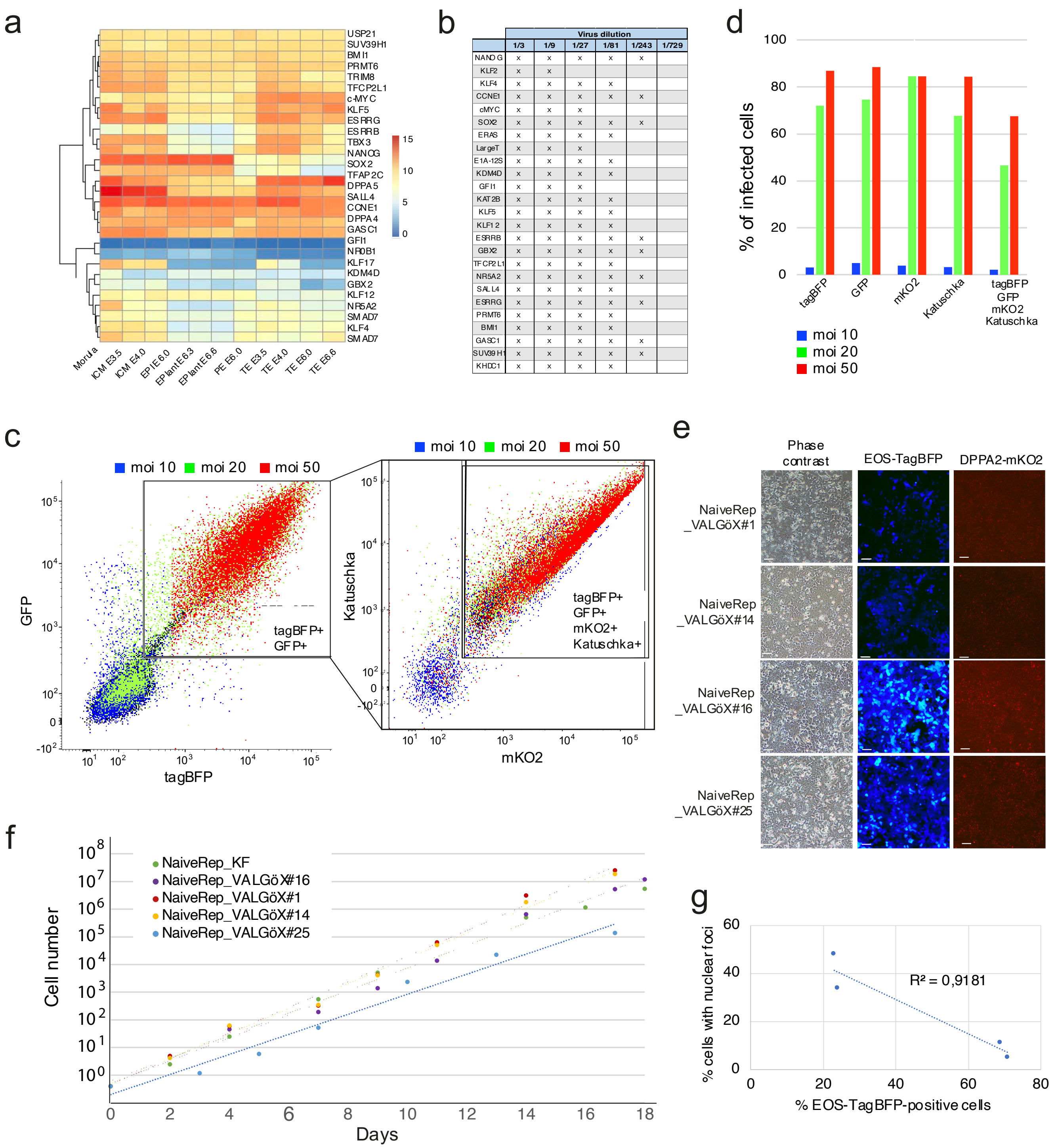

	Virus dilution					
	1/3	1/9	1/27	1/81	1/243	1/729
NANOG	x	x	x	x	x	
KLF2	x	x				
KLF4	x	x	x	x		
CCNE1	x	x	x	x	x	
cMYC	x	x	x			
SOX2	x	x	x	x	x	
ERAS	x	x	x	x		
LargeT	x	x	x			
E1A-12S	x	x	x	x		
KDM4D	x	x	x	x		
GFI1	x	x	x			
KAT2B	x	x	x	x		
KLF5	x	x	x	x		
KLF12	x	x	x	x		
ESRRB	x	x	x	x	x	
GBX2	x	x	x	x	x	
TFCP2L1	x	x	x	x		
NR5A2	x	x	x	x	x	
SALL4	x	x	x	x		
ESRRG	x	x	x	x	x	
PRMT6	x	x	x	x		
BMI1	x	x	x	x		
GASC1	x	x	x	x	x	
SUV39H1	x	x	x	x	x	
KHDC1	x	x	x	x		

Supplementary Figure 2: cDNA library screening. (**a**) Heatmap representation of the expression in the rabbit embryo of the 29 genes included in the cDNA library. ICM, inner cell mass; E, embryonic day; EPI, epiblast; EPIant, anterior epiblast; PE, primitive endoderm; TE, trophectoderm. (**b**) Estimation of relative lentivirus titers by detection of proviral DNA using genomic PCR in B19_KF cells transduced with each of the 25 corresponding lentiviruses at the indicated dilution. (**c**) Flow cytometry analysis of B19_KF cells transduced with four lentiviral vectors, each expressing a reporter fluorescent protein (i.e. GFP, mKO2, tagBFP, and Katushka) at multiplicities of infection (MOI) of 10, 20 and 50 (1:1:1:1 ratio). (**d**) Percentage of B19_KF cells transduced with each one of the four reporter lentiviruses and with all. (**e**) Phase contrast and tagBGP/mKO2 epifluorescence images of NaiveRep_VALGöX cell lines. Scale bars, 50 µm. (**f**) Growth curves of NaiveRep_KF and NaiveRep_VALGöX cell lines. (**g**) Negative correlation between percentage of cells with nuclear foci and percentage of cells expressing EOS-tagBFP in NaiveRep_VALGöX cell lines.

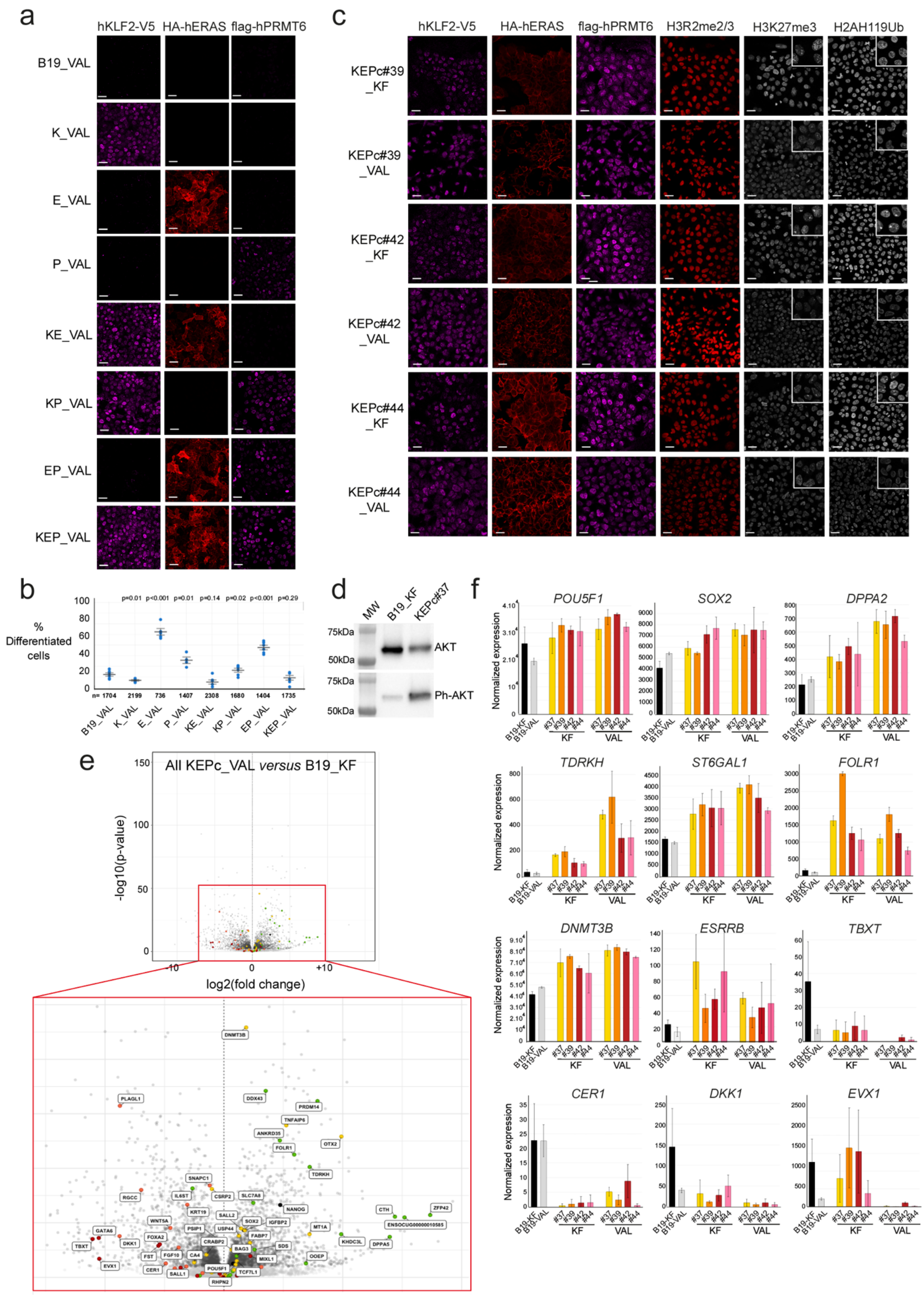

Supplementary Figure 3: Characterization of KEPc cells (**a**) Confocal imaging of B19_VAL cells before and after single, double, or triple transfection with PiggyBac plasmids expressing KLF2-V5 (K), HA-ERAS (E), and Flag-PRMT6 (P). Immunostaining was performed using anti-V5 (KLF2), anti-HA (ERAS), and anti-Flag (PRMT6) antibodies. Scale bars, 40 µm. (**b**) Percentage of differentiated cells in control, single-, double-, and triple-transfected cell populations, based on fluorescence patterns (diffuse vs punctate) observed

following H3K27me3 immunostaining. H3K27me3 fluorescence intensity was quantified in individual cells (n indicates the number of cells analyzed). Each dot represents the mean percentage of differentiated cells per replicate. Error bars denote mean ± SEM. Comparisons with B19_VAL conditions were performed using a two-sided Welch's t-test (p-values indicated above each chart). (**c**) Confocal imaging of immunostaining for KLF2-V5, HA-ERAS, Flag-PRMT6, H3R2me2/3, H3K27me3, and H2AK119Ub in control B19_VAL, KEPc#39, KEPc#42, and KEPc#44 lines cultured under _KF and _VAL conditions (3 independent replicates). Scale bars, 30 µm. (**d**) Western blot analysis of total AKT and phosphorylated AKT (Ph-AKT) in B19_KF and KEP#37_KF cells. (**e**) Volcano plot showing differentially expressed genes between B19_KF and all KEPc_VAL cell lines. (**f**) Histograms of normalized gene expression in B19_KF, B19_VAL_48h, KEPc_KF, and KEPc_VAL cell lines based on RNA-seq data). Mean values and standard deviations were calculated from three independent replicates.

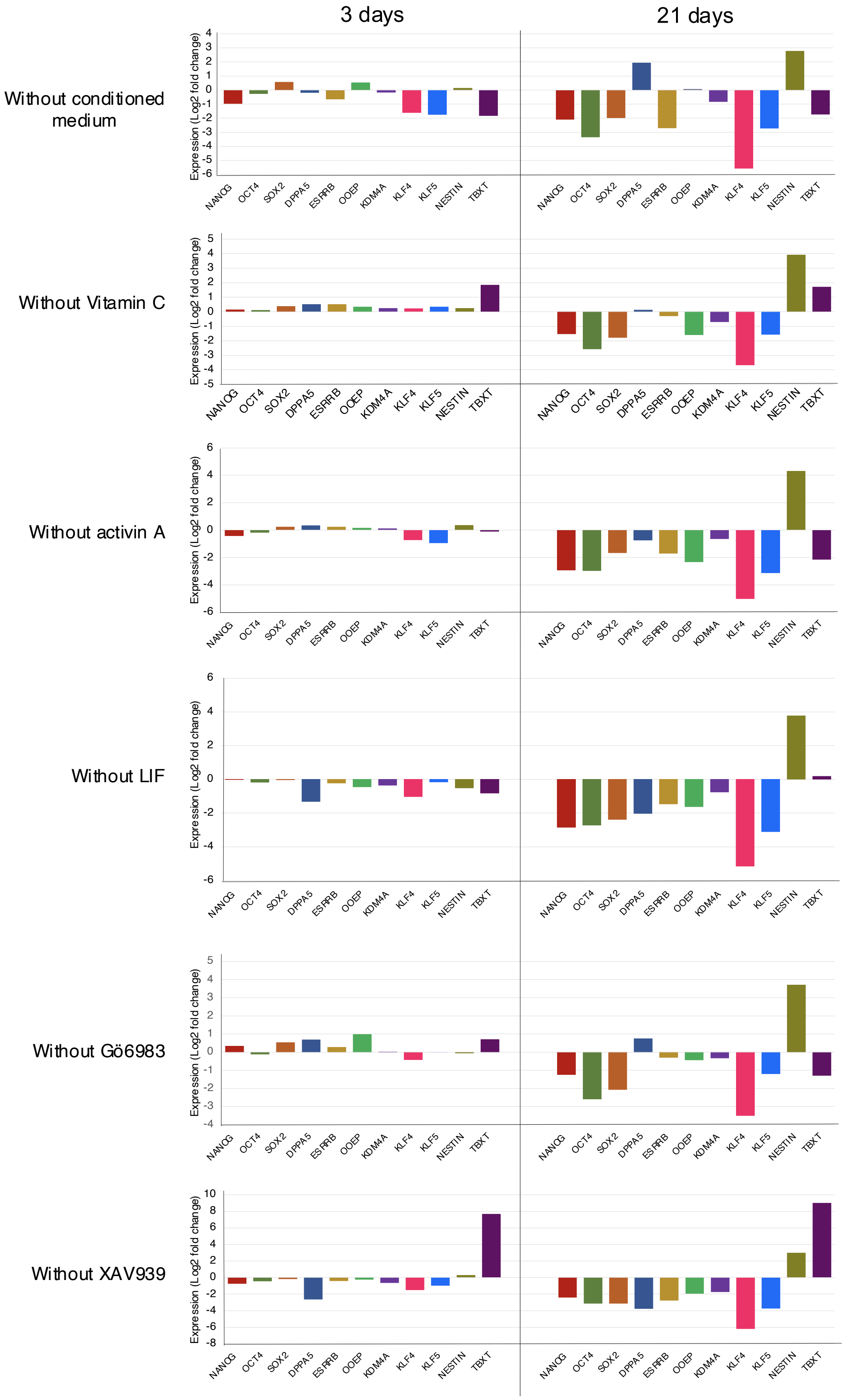

Supplementary Figure 4: Synergy between transgenes and VALGöX. qRT-PCR analysis of gene expression in KEPc#37_VAL cells after withdrawal of each individual component of the VALGöX medium for 3 and 21 days, and compared to control KEPc#37_VAL cells.

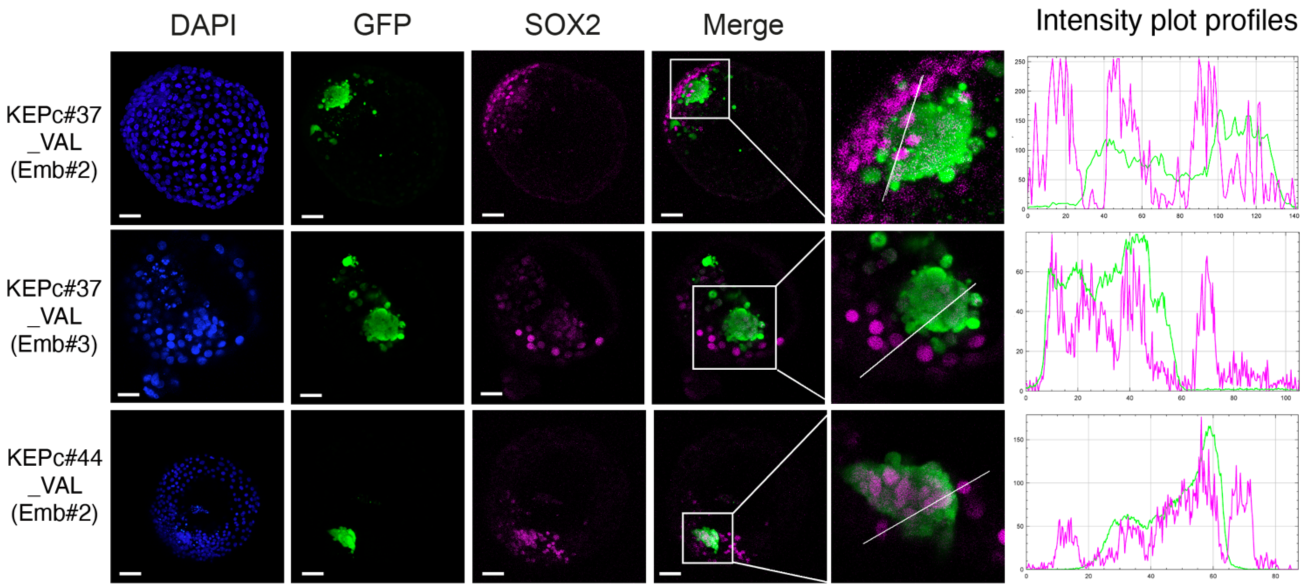

Supplementary Figure 5: Colonization of rabbit embryos by iPSCs overexpressing KLF2, ERAS and PRMT6. Confocal images of late-blastocyst-stage rabbit embryos (E5.0, 3DIV) acquired after the microinjection of KEPc#37_VAL and KEPc#44_VAL cells into early morula-stage (E2.8) embryos (scale bars: 50 µM). On the right part of the panel, a single line has been drawn through the ICM of the embryos for further intensity profile measurement using Fiji. The resulting fluorescence intensity plot profile across multiple channels (green for GFP signal, purple for SOX2 signal) demonstrates that GFP cells are also $SOX2^{+}$. The embryos shown are representative of three independent experiments performed for each cell line and condition

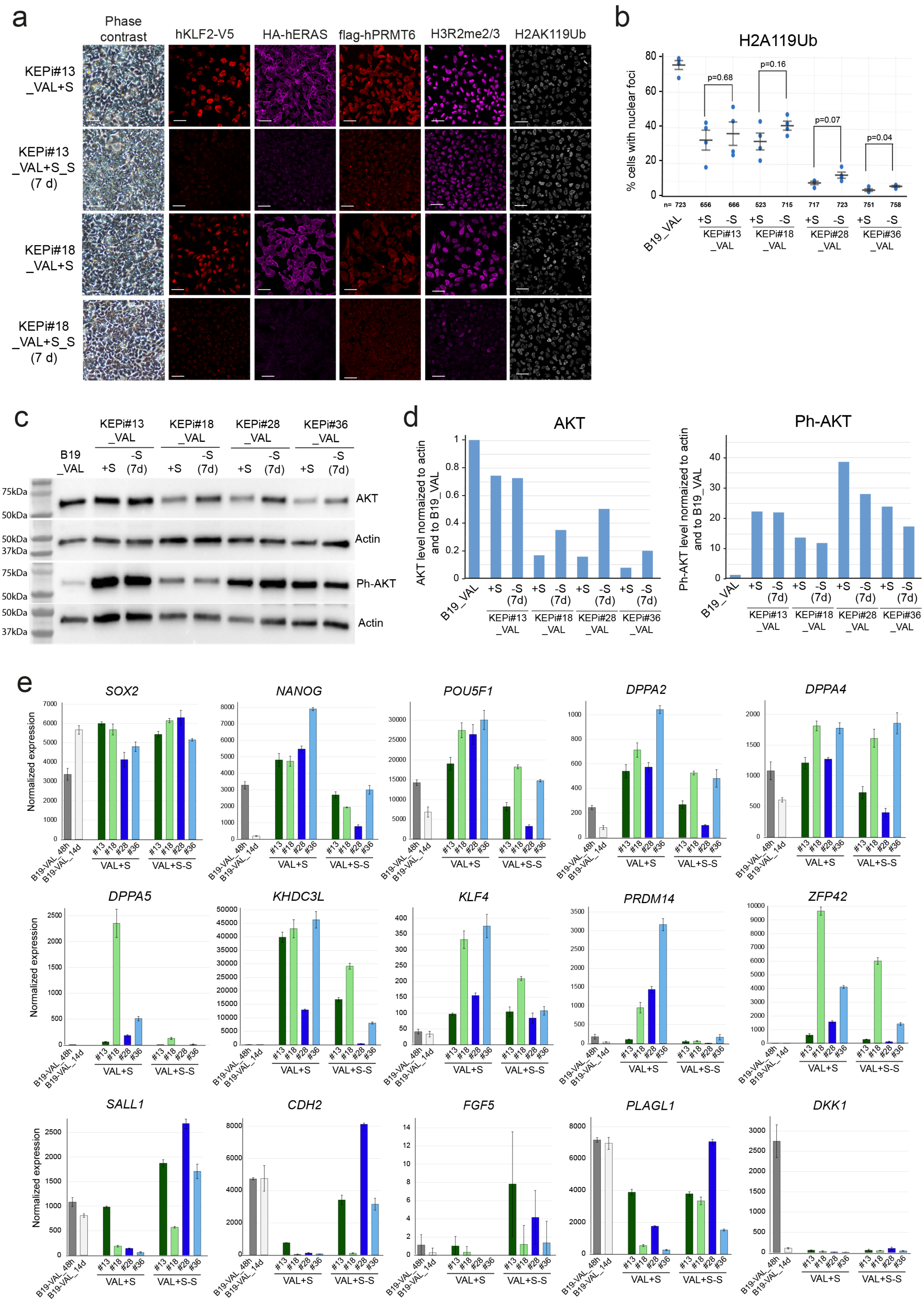
a
Phase contrast
hKLF2-V5
HA-hERAS
flag-hPRMT6
H3R2me2/3
H2AK119Ub
KEPi#13 _VAL+S
KEPi#13 _VAL+S_S (7 d)
KEPi#18 _VAL+S
KEPi#18 _VAL+S_S (7 d)
b
H2A119Ub
% cells with nuclear foci
p=0.68
p=0.16
p=0.07
p=0.04
n= 723 656 666 523 715 717 723 751 758
B19_VAL
+S -S +S -S +S -S +S -S
KEPi#13 _VAL
KEPi#18 _VAL
KEPi#28 _VAL
KEPi#36 _VAL
c
B19 _VAL
KEPi#13 _VAL
KEPi#18 _VAL
KEPi#28 _VAL
KEPi#36 _VAL
+S -S (7d)
75kDa
50kDa
37kDa
AKT
Actin
Ph-AKT
d
AKT
AKT level normaized to actin and to B19_VAL
Ph-AKT
Ph-AKT level normalized to actin and to B19_VAL
e
SOX2
NANOG
POU5F1
DPPA2
DPPA4
DPPA5
KHDC3L
KLF4
PRDM14
ZFP42
SALL1
CDH2
FGF5
PLAGL1
DKK1
Normalized expression
B19-VAL_48h
B19-VAL_14d
#13 #18 #28 #36
VAL+S
VAL+S-S

Supplementary Figure 6: Characterization of KEPi cells. (**a**) Confocal imaging of KLF2-V5, HA-ERAS, flag-PRMT6, H3R2me2/3, and H2AK119Ub immunostaining in KEPi#13_VAL+S, KEPi#13_VAL+S-S, KEPi#18_VAL+S, and KEPi#18_VAL+S-S cells (n = 2 independent experiments). Scale bars: phase contrast, 100 µm; immunofluorescence images, 30µm. (**b**) Histograms showing the percentage of cells with H2AK119Ub nuclear foci in B19_VAL, KEPi#13_VAL+S, KEPi#13_VAL+S-S, KEPi#18_VAL+S, and KEPi#18_VAL+S-S cells. Each cell type was quantified independently (exact n indicated) from four biological replicates. Dots represent the mean percentage of cells with nuclear foci per replicate. Error bars represent the mean ± SEM. Comparisons between +S and –S conditions were made using a two-sided Welch's t-test (p-values indicated above each chart). (**c**) Western blot analysis of total AKT and phosphorylated AKT (Ph-AKT) in B19_VAL, KEPi#13_VAL+S, KEPi#13_VAL+S-S, KEPi#18_VAL+S, and KEPi#18_VAL+S-S cells, with ACTIN used as a loading control. (**d**) Histogram showing normalized levels of AKT and Ph-AKT in KEPi# cells cultured under +S and +S-S conditions for 7 days. Values were normalized to ACTIN and to B19_VAL reference levels. Quantification of Western blot bands was performed using Fiji with the Gel Analyzer macro (n = 1 experiment).. (**e**) Histogram showing normalized expression of selected genes in B19_VAL_48h, B19_VAL_14d, KEPi#_VAL+S, and KEPi#_VAL+S-S cells (RNA-seq data). Means ± SD are shown from three independent replicates.

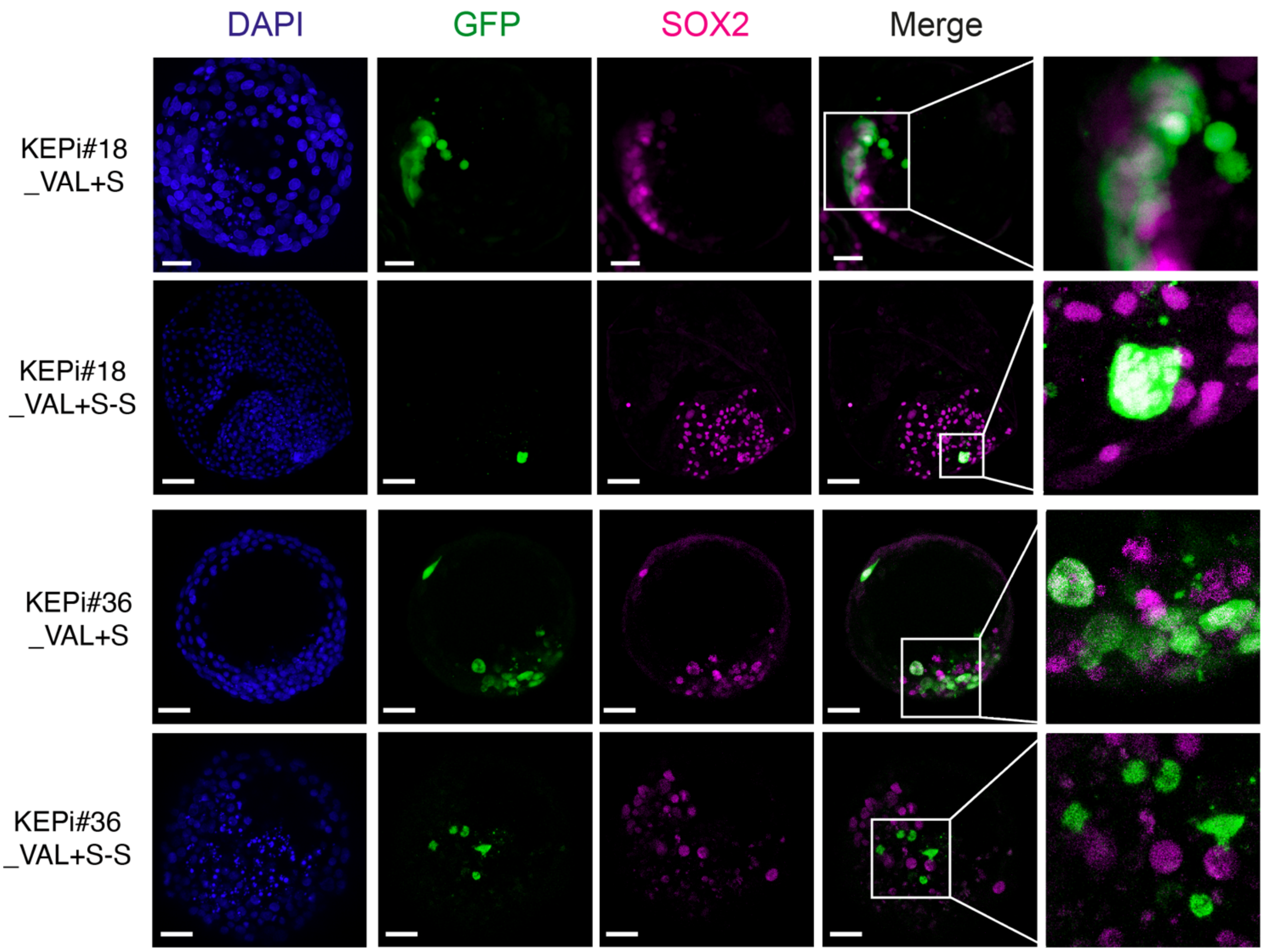

Supplementary Figure 7: Design of a reversible KEP expression system for *in vivo* studies. Confocal images of late-blastocyst-stage rabbit embryos (E5.0, 3DIV) following microinjection of KEPi_VAL+S and KEPi_VAL+S-S cells into morula-stage embryos (E2.8). Scale bars, 50 µm. (**f**) Percentage of embryos containing GFP$^+$ cells for each condition. Data are representative of three independent experiments performed per cell line and culture condition.

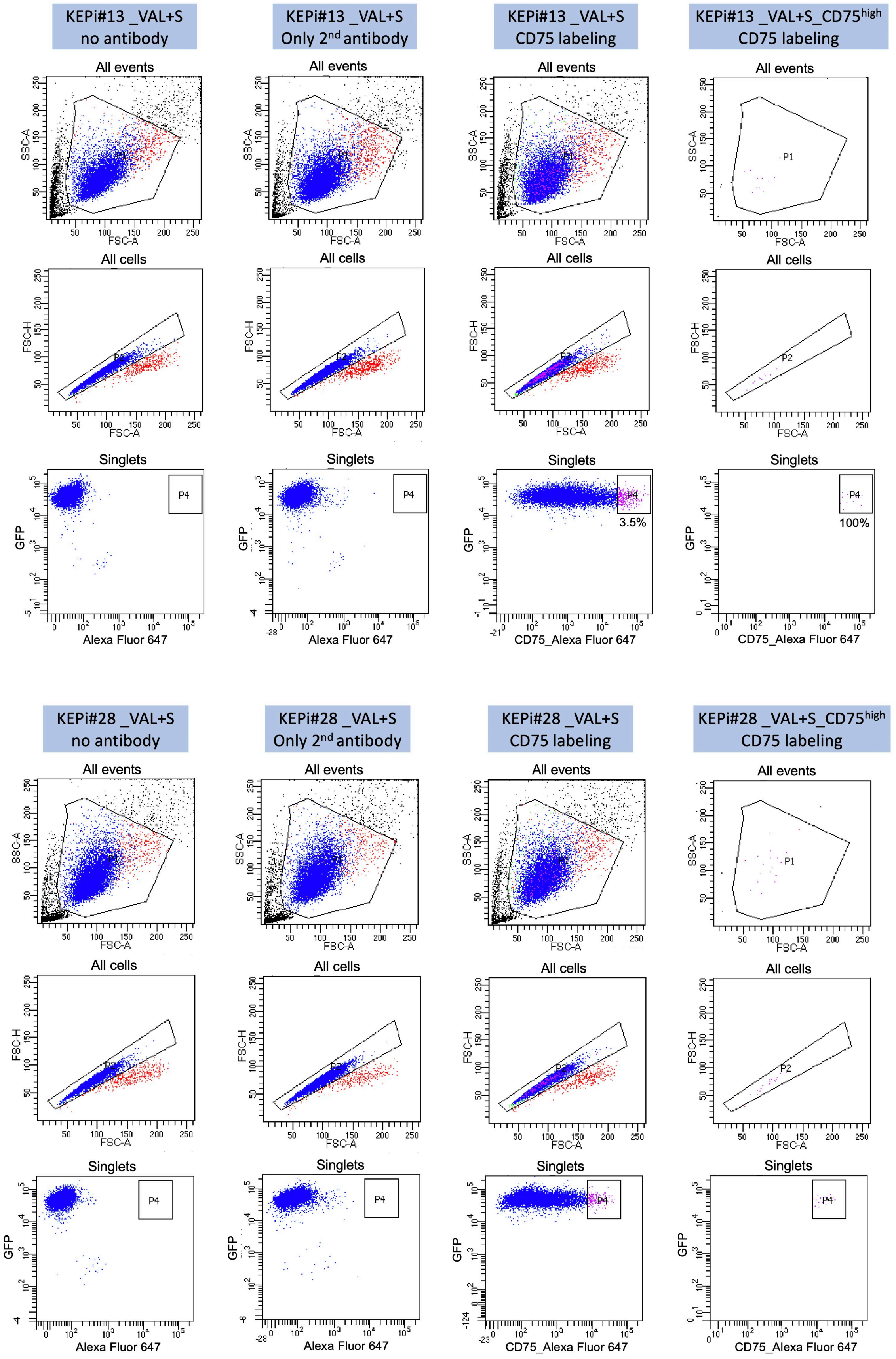

Supplementary Figure 8: FACS sorting of CD75^{high} cells from KEP#13_VAL+S and KEP#28_VAL+S cells. Total cells were gated based on FSC-A and SSC-A. Single cells were selected using FSC-H / FSC-A gating. "No antibody" and "no primary antibody" controls were employed to identify any unspecific labeling.

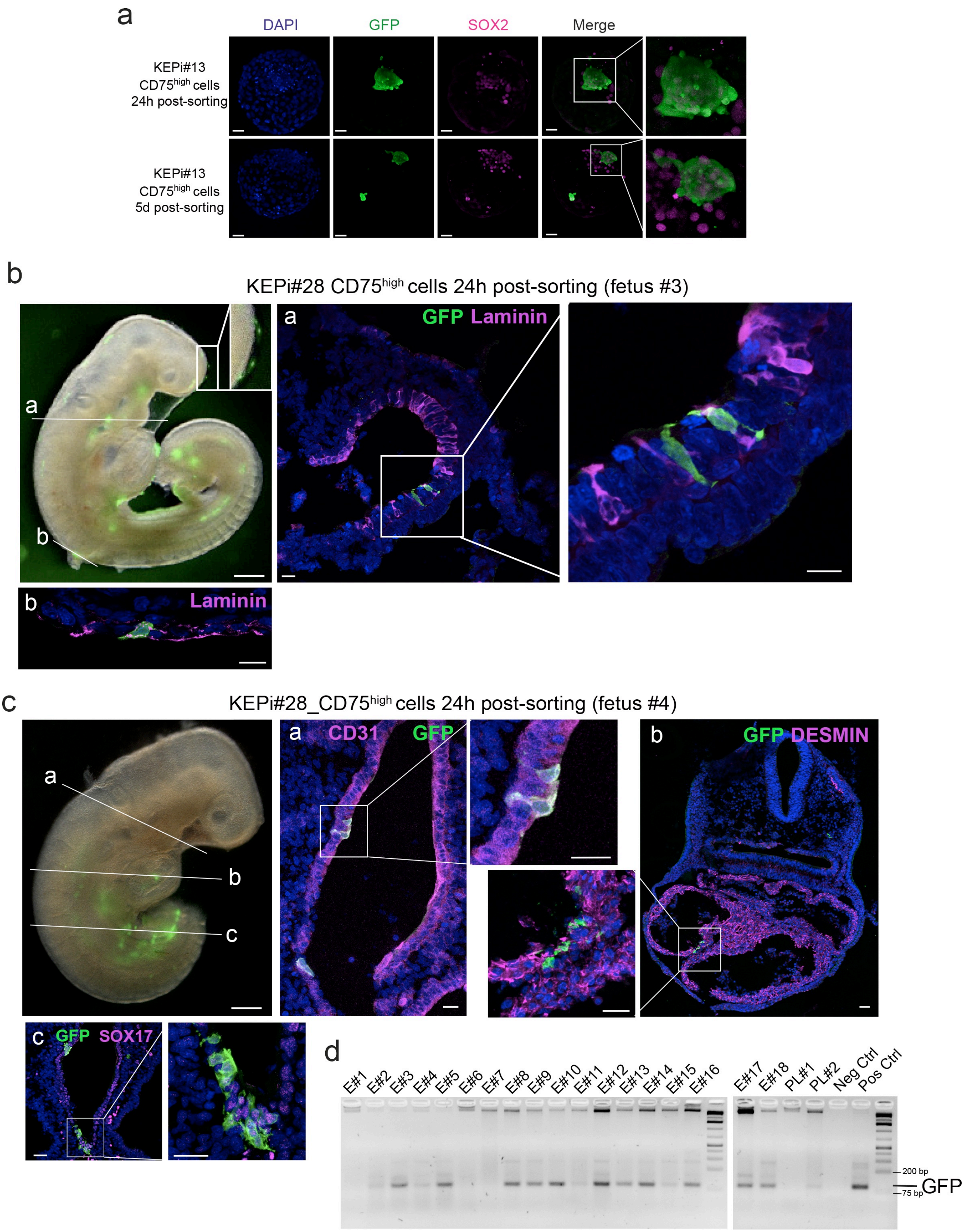

Supplementary Figure 9: Colonization of rabbit embryos and fetuses by KEPi_CD75^{high} cells. (a) Confocal images of late-blastocyst-stage rabbit embryos (E5.0, 3 DIV) following microinjection of KEPi#13_ CD75^{high} cells into morula-stage embryos (E2.8). Three independent experiments were performed. Scale bars: 50 µm). (**b**) Chimeric fetus (#3) collected at E10.5, showing robust contribution of KEPi#28_ CD75^{high} cell-derivatives in the amnion, surface ectoderm, and gut epithelium (co-labeled with LAMININ). Scale bar, 200 µm (whole fetus), 20 µm (sections). (**c**) Chimeric fetus (#4) collected at E10.5, showing high contribution of

KEPi#28_ CD75^{high} cell-derivatives in the gut epithelium (co-labeled with CD31 and SOX17), and in cardiac cells (co-labeled with DESMIN). Scale bar, 200 µm (whole fetus), 20 µm (sections). Three technical replicates were performed. (**d**) Gel electrophoresis of PCR products following amplification of GFP DNA from genomic DNA extracted from E10.5 fetuses (#1 to #18) and placentas (PL#1, PL#2). DNA from a wild-type rabbit was used as a negative control; plasmid DNA carrying *GFP* was used as a positive control.

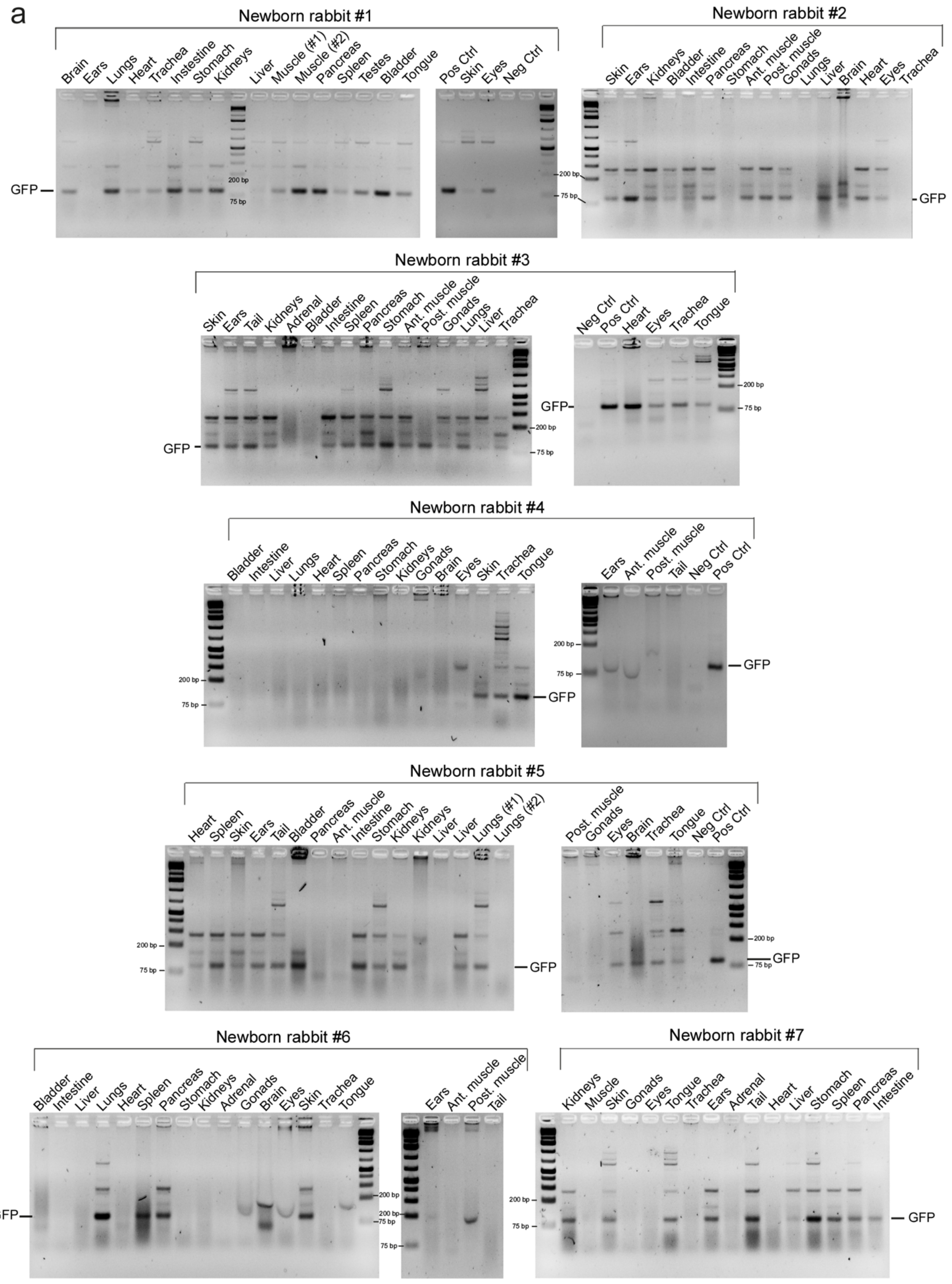

Supplementary Figure 10: PCR analysis of KEPi_CD75^{high} cell contribution in newborns. Gel electrophoresis of PCR fragments after amplification of a GFP DNA sequence from genomic DNA extracted from organs of newborns (stillborn or sacrificed), with DNA of wild-type rabbit as negative control and DNA of a plasmid carrying GFP gene.

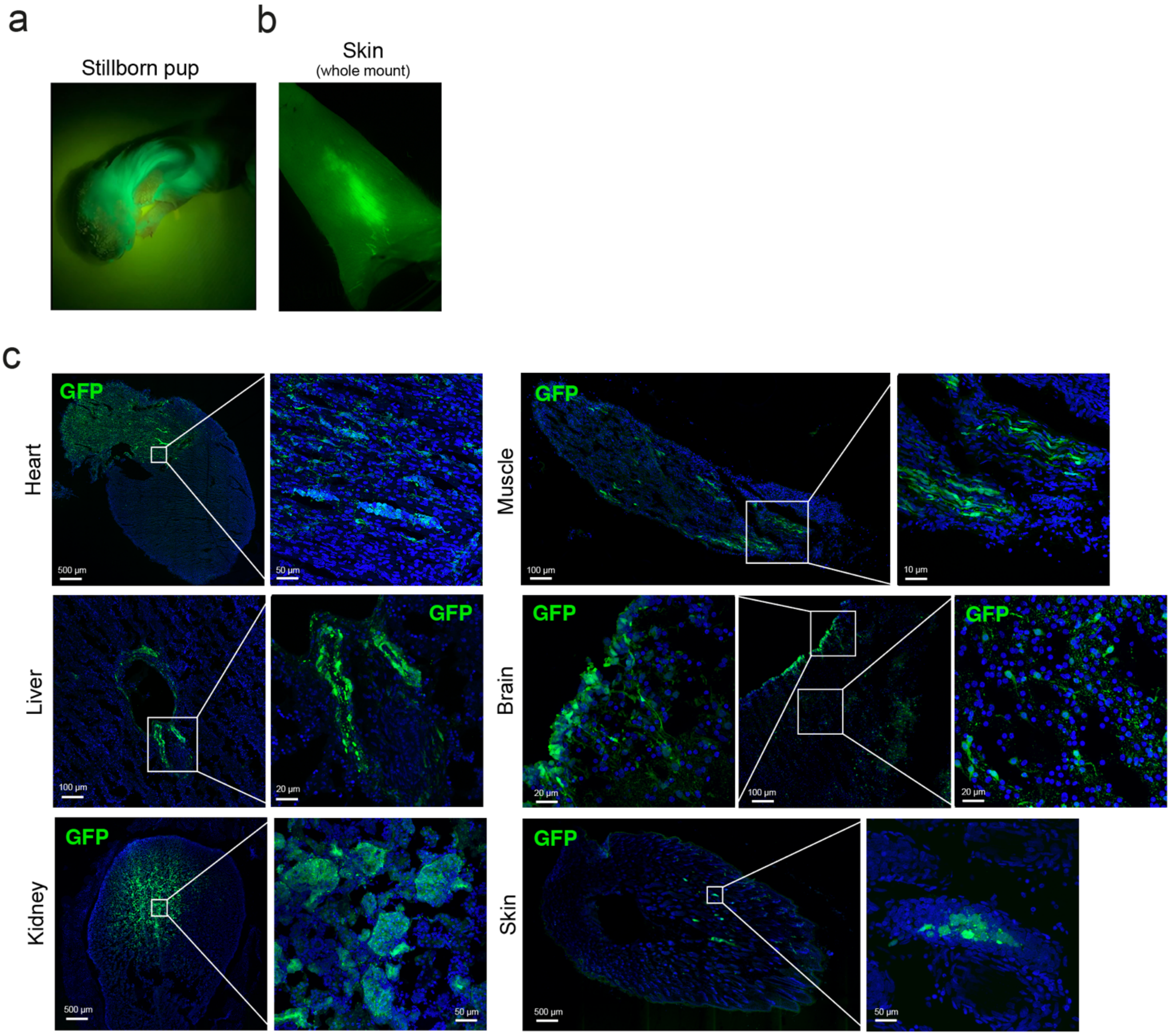

Supplementary Figure 11: Contribution of KEPi_CD75^{high} cells to organ development in newborns. (**a**) Whole stillborn pup photographed under UV light. (**b**) Isolated piece of skin imaged under UV light. (**c**) Confocal microscopy images showing GFP$^+$ cells labelled with anti-GFP antibody in tissue sections from a newborn chimera sacrificed one day after birth (3 technical replicates).

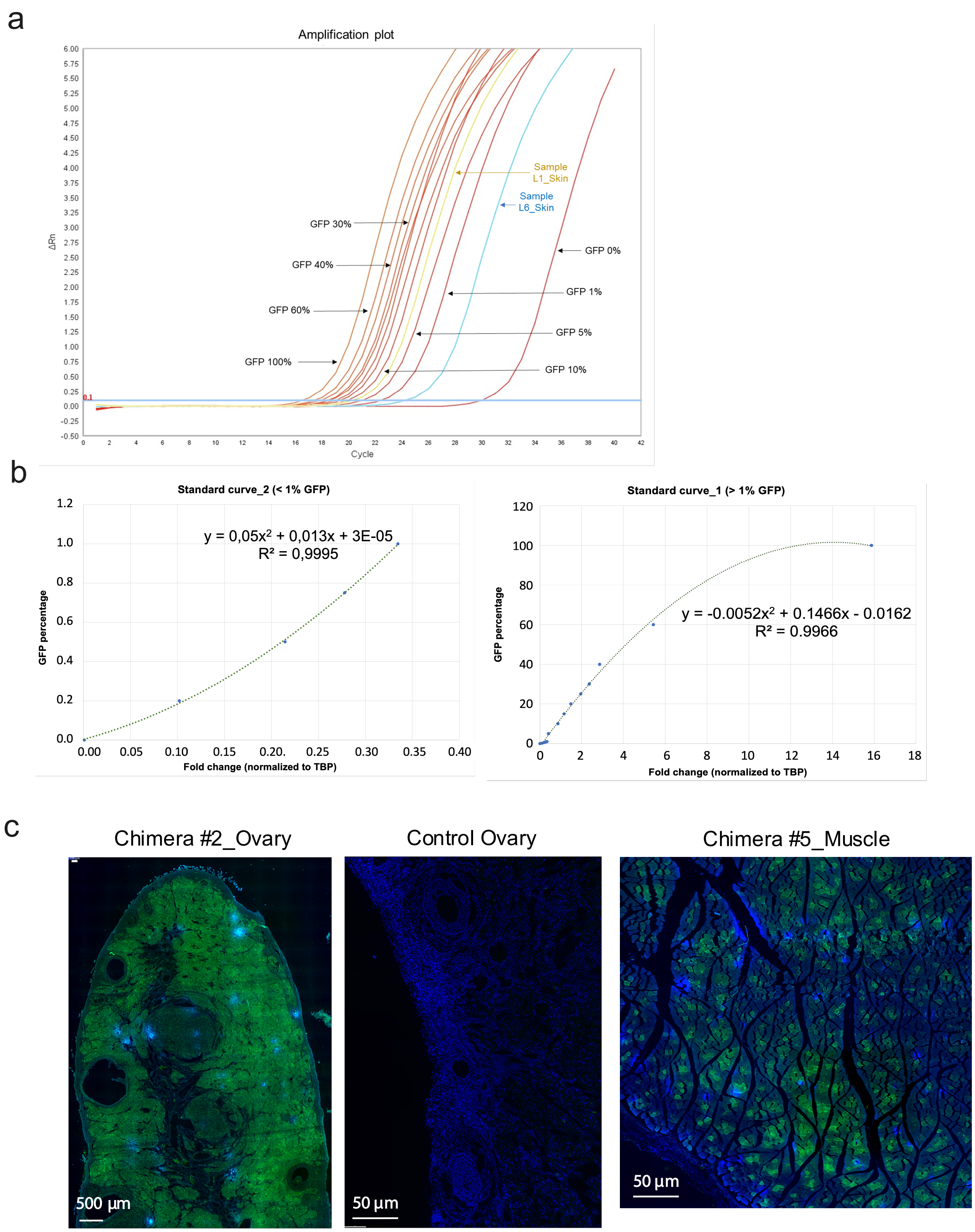

Supplementary Figure 12: qPCR analysis of KEPi_CD75^{high} cell contribution in adults. (**a**) Amplification plots generated from samples containing defined percentages of GFP DNA used to construct standard curves. (**b**) GFP standard curves established for quantifying GFP DNA levels. (**c**) Widefield epifluorescent images, with deconvolution, showing GFP^{+} cells labelled with anti-GFP antibody in tissue sections from adult female chimeras #A2 (ovary) and #A5 (muscle). A wild-type rabbit is shown as a negative control for immunostaining.

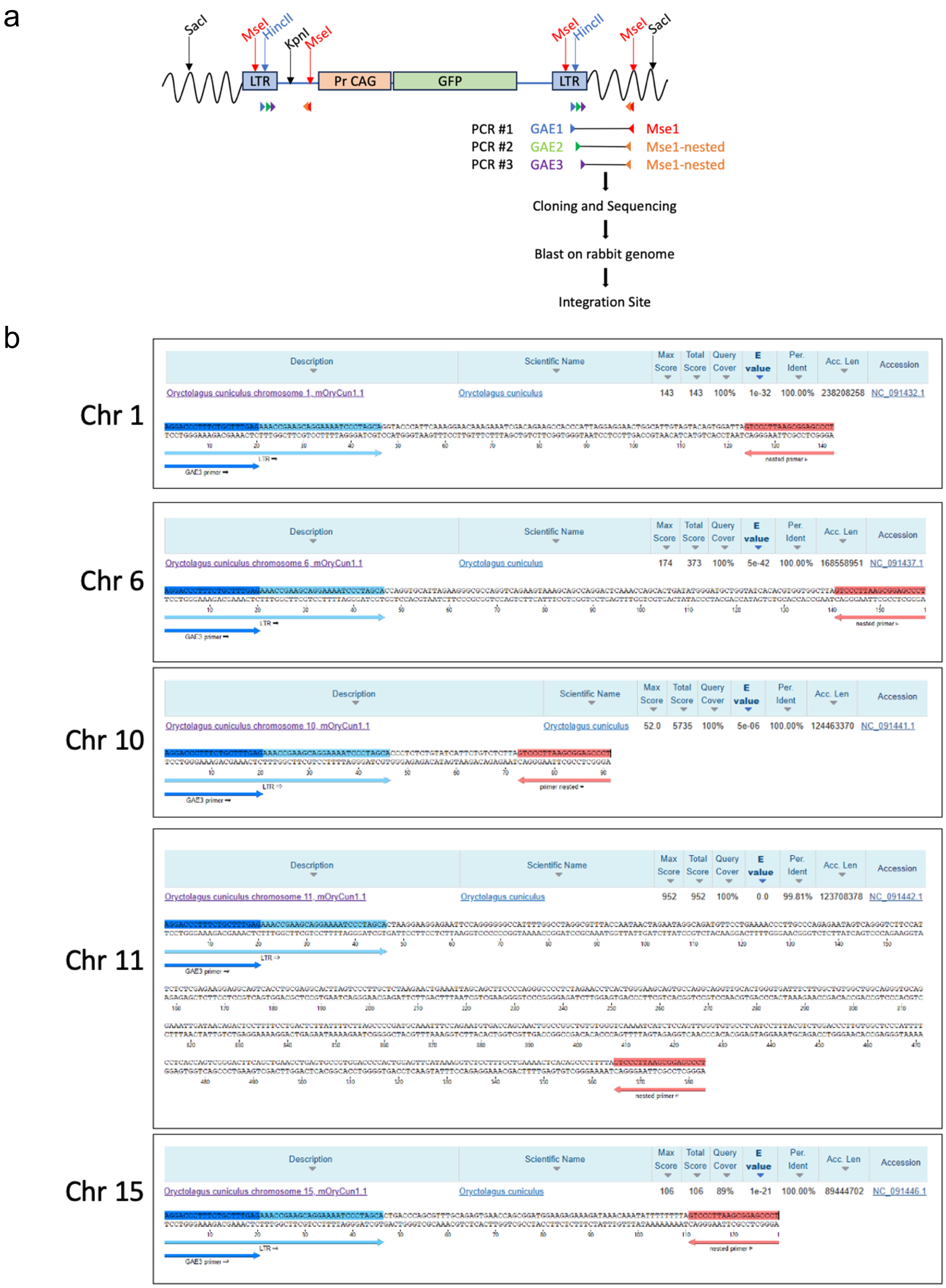

Supplementary Figure 13: Identification of proviral DNA integration sites in KEPi#28 cells. (**a**) Schematic representation of the proviral structure and the PCR strategy used to amplify genomic DNA fragments flanking integration sites. (**b**) Mapping of integration sites within the rabbit genome using BLAT (UCSC Genome Browser, *OryCun2* assembly; Rabbit, April 2009, Broad Institute) and BLAST (NCBI, *mOryCun1.1* assembly).

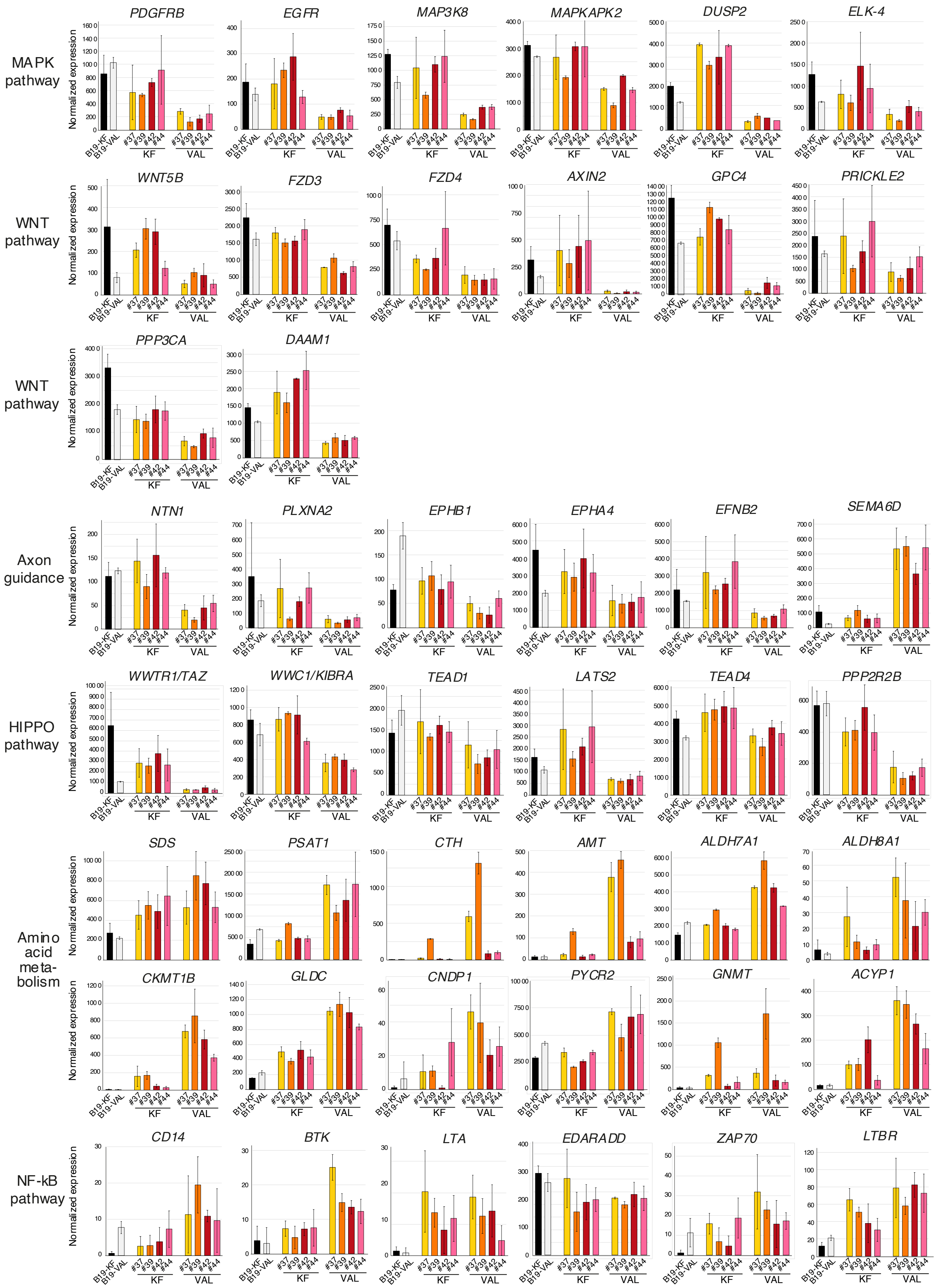
MAPK pathway
PDGFRB
EGFR
MAP3K8
MAPKAPK2
DUSP2
ELK-4
WNT pathway
WNT5B
FZD3
FZD4
AXIN2
GPC4
PRICKLE2
WNT pathway
PPP3CA
DAAM1
Axon guidance
NTN1
PLXNA2
EPHB1
EPHA4
EFNB2
SEMA6D
HIPPO pathway
WWTR1/TAZ
WWC1/KIBRA
TEAD1
LATS2
TEAD4
PPP2R2B
Amino acid meta-bolism
SDS
PSAT1
CTH
AMT
ALDH7A1
ALDH8A1
CKMT1B
GLDC
CNDP1
PYCR2
GNMT
ACYP1
NF-kB pathway
CD14
BTK
LTA
EDARADD
ZAP70
LTBR
Normalized expression
B19-KF
B19-VAL
#37
#39
#42
#44
KF
VAL

Supplementary Figure 14: Transcriptome characterization of KEPc cells. Histogram showing normalized expression levels of selected genes in B19_KF, B19_VAL_48h, KEPc_KF, and KEPc_VAL cells, based on RNA-seq data. Values represent means ± standard deviations from three independent replicates. Gene categories include:

- **MAPK signaling pathway:** Receptor-encoding genes *PDGFRB*, *EGFR*; signaling molecules *MAP3K8*, *MAPKAPK2*; phosphatase *DUSP2*; and downstream target *ELK4*.
- **WNT signaling pathway:** Ligands and modulators *WNT5B*, *GPC4*; receptors *FZD3*, *FZD4*; adaptors *PRICKLE2*, *AXIN2*, *DAAM1*; and *PPP3CA*, encoding calcineurin A (non-canonical WNT/Ca^{2+} pathway).
- **Axon guidance:** *EFNB2* (Ephrin-B2); receptors *EPHB1*, *EPHA4*; guidance cues *NTN1* (Netrin-1) and *PLXNA2* (Plexin A2).
- **HIPPO signaling pathway:** Regulators *WWTR1/TAZ*, *WWC1/KIBRA*, *LATS2*, *PPP2R2B*; transcriptional targets *TEAD1* and *TEAD4*.
- **Amino acid metabolism:** Enzyme-encoding genes involved in serine, glycine, lysine, cysteine, alanine, arginine, methionine, proline, tryptophan, and glutamate homeostasis: *SDS*, *CTH*, *GLDC*, *PSAT1*, *AMT*, *ALDH7A1*, *ALDH8A1*, *GNMT*, *CNDP1*, *CKMT1B*, *A CYP1*, *PYCR2*. These genes also regulate pyruvate production (*SDS*), one-carbon metabolism (*SDS*, *GLDC*, *PSAT1*, *GNMT*), and energy metabolism (*CKMT1B*).
- **NF-κB signaling pathway:** *BTK*, *EDARADD*, *ZAP70*, *LTBR*, and *CD14*, encoding factors that activate IKK degradation and NF-κB signaling.

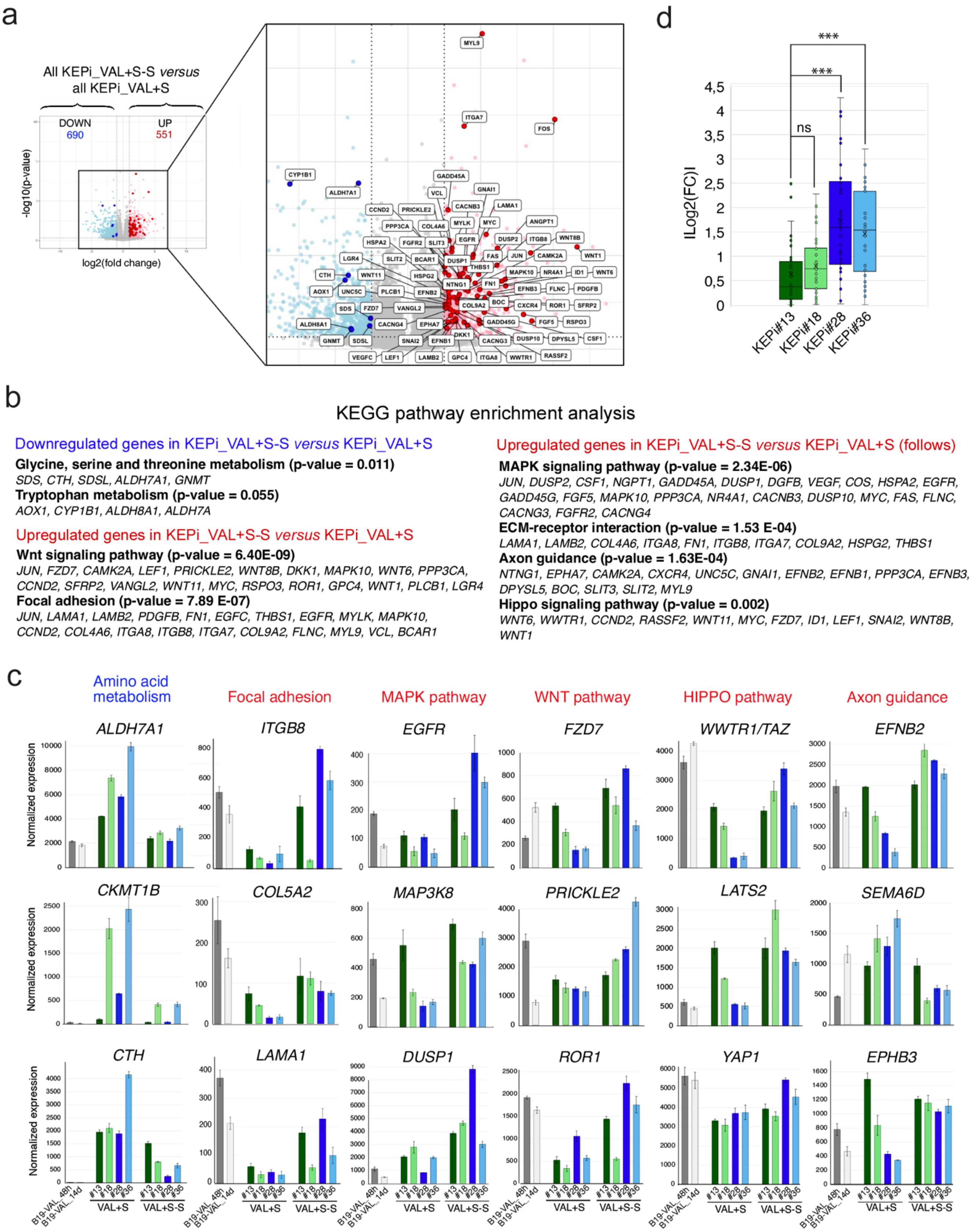

Supplementary Figure 15: Transcriptome characterization of KEPi_VAL cells. (**a**) Volcano plot showing differentially expressed genes (DEGs) between all KEPi_VAL+S and all KEPi_VAL+S-S cell lines. (**b**) KEGG pathway enrichment analysis of DEGs between KEPi_VAL+S-S and KEPi_VAL+S cells. (**c**) Histogram showing normalized expression levels of selected genes in B19_KF, B19_VAL, KEPi_VAL+S, and KEPi_VAL+S-S cells. Data represent means ± standard deviations from three independent replicates. (**d**) Box plot of average fold-changes calculated from DEGs between each KEPi_VAL+S and KEPi_VAL+S-S

cell pair (Mann-Whitney U test; ***, $p<0.001$). Means are indicated by crosses; boxes represent the median and interquartile range; whiskers show the full data range. Points beyond the whiskers are considered potential outliers.

Supplementary Table 1: Results of chimeric newborn analysis based on GFP-associated fluorescence and the presence of the GFP gene in the organs or tissues of the seven chimeric newborns.

	Newborn 1		Newborn 2		Newborn 3		Newborn 4		Newborn 5		Newborn 6		Newborn 7	
Age Of Death	1 day		2 days		1 day		1 day		1 day		1 day		5 days	
Cause Of Death	Natural		Sacrificed		Natural		Sacrificed		Sacrificed		Sacrificed		Sacrificed	
Sex$	Male		Female		Male		Male		Male		Female		Male	
Organs/Tissues	Fluo*	PCR£	Fluo*	PCR£	Fluo*	PCR£	Fluo*	PCR£	Fluo*	PCR£	Fluo*	PCR£	Fluo*	PCR£
Brain	+	+	-	+	+	+	-	-	+	+	-	-	-	-
Ears	-	-	-	+	+	+	-	+	+	+	-	-	+	+
Lungs	+	+	-	-	-	+	-	-	+	+	-	+	-	+
Heart	+	+	-	+	+	+	-	-	+	+	-	-	-	-
Trachea	+	+	-	-	+	+	-	+	+	+	-	-	-	-
Intestine	-	+	-	+	+	+	-	-	+	+	-	-	-	+
Stomach	+	+	-	-	+	+	-	-	+	+	-	-	+	+
Kidneys	+	+	+	+	-	+	-	-	-	+	-	-	+	+
Spleen	+	+	N.D.		-	+	-	-	+	+	-	+	-	+
Liver	+	+	+	+	+	+	-	-	+	+	-	-	+	+
Anterior Muscle	+	+	-	+	+	+	-	-	-	-	-	-	-	-
Posterior Muscle	+	+	-	+	-	+	-	+	-	-	+	+	-	-
Pancreas	-	+	-	+	-	+	-	-	-	-	+	+	-	+
Gonads	+	+	-	+	-	+	-	-	-	-	-	-	-	-
Bladder	+	+	+	+	-	-	-	-	+	+	-	-	-	-
Tongue	+	+	-	+	+	+	-	+	+	+	-	-	+	+
Skull Skin	+	+	+	+	-	+	-	+	-	+	+	+	+	+
Tail	N.D.		N.D.		-	+	-	-	-	+	-	-	-	+
Eyes	+	+	-	+	+	+	-	-	+	+	+	+	-	-

* Fluorescence detection: **+** = GFP positive; **-** = GFP negative.
£ PCR detection of GFP gene: **+** = presence; **-** = absence;
$ Determined after examination of testes vs ovaries
N.D. = Not Determined

Supplementary Table 2: Quantitative PCR analysis of GFP DNA in various organs of adult chimeras

Tissue	Cerebellum	Heart	Intestine	Kidney left	Kidney right	Liver	Lung	Muscle	Pancreas	Skin	Spleen
**Rabbit #1**	0.02%	6.91%	0.63%	0.09%	0.93%	3.43%	0.1%	64.84%	100%	22.73%	0.01%
**Rabbit #2**	0.02%	0.02%	0.02%	5.36%	18.21%	11.22%	33.2%	57.06%	35.18%	100%	9.26%
**Rabbit #3**	1.01%	0.1%	0.04%	0.02%	0.13%	0.09%	0.05%	0.32%	0.53%	0.04%	0.03%
**Rabbit #4**	0.01%	0.01%	0.01%	0.01%	0.05%	0.01%	0.01%	0.07%	< 0.01%	0.08%	0.01%
**Rabbit #5**	NA	99.82%	15.45%	0.07%	0.11%	0.19%	0.2%	88.66%	0.03%	0.09%	0.04%
**Rabbit #6**	0.29%	0.03%	0.01%	0.02%	0.02%	0.02%	18.97%	0.01%	0.01%	0.14%	< 0.01%

NA, not analyzed

Supplementary Table 3: Origin and amplification of cDNAs included in the cDNA library.

cDNA	Origin	Primers
eGFP	pW10-eGFP (Negre et al., 2000)	-
mKO2	pFucci-G_1 Orange (MBL Life Science, AM-V9003M)	F: 5'- GCTACCGGTCGCCACCATGGTGAGTGTG-3'<br>R: 5'-ATTGAACTCGAGCTAGTAATGAGCTACTGCATCTTCTACC -3'
tagBFP	pCAG-mtagBFP (addgene #89685)	F: 5'- ATGAGCGAGCTGATTAAGG-3'<br>R: 5'- CTTAATTAAGCTTG -3'
Kate2	pMA-RQ-FGF5-KAT-FAB (Lifetechnology)	F: 5'- GCTACCGGTCGCCACCATGGTTGGAGAGGACTCTG-3'<br>R: 5'- ATTGAACTCGAGTCAGGAATGTCCCAACTTG-3'
BMI1	pT3-EF1a-Bmi1 (Addgene #31783)	F: 5'-AACACAGGTGTCGTGACGCGATGCATCGAACAACGAGAATC-3'<br>R: 5'-GCTAGCTACTAGCTAGTCGATCAACCAGAAGAAGTTGC -3'
CCNE1	pHCAGhcycE1 (Coronado et al., 2013)	F: 5'-GCTACCGGTCGCCACCATGCCGAGGGAGCGCAGGGAGCGGGATG CGAAGGAGCGG -3'<br>R: 5'- ATTGAACTCGAGTCACGCCATTTCCGGCC -3'
DAX1	pAd/CMV/DAX1-IRES-nEGFP (Addgene#29752)	F: 5'- GCTACCGGTCGCCACCATGGCGGGCGAGAACCACCAGTG-3'<br>R: 5'-ATTGAACTCGAGTTATATCTTTGTACAGAGCATTTCCAGCATC-3'
DPPA2	RT-PCR from human PSCs	F: 5'- GCTACCGGTCGCCACCATGTCAGATGCAAATTTGGATAG-3'<br>R: 5'- ATTGAACTCGAGCTACTTCTCTACTGTCATTAATC-3'
DPPA4	RT-PCR from human PSCs	F: 5'- GCTACCGGTCGCCACCATGTTGCGAGGCTCCGC-3'<br>R: 5'- ATTGAACTCGAGCTATTCCCATTGGAGGCTTTTATTAAGACC-3'
DPPA5	RT-PCR from human PSCs	F: 5'- GCTACCGGTCGCCACCATGGGAACTCTCCCGGCA-3'<br>R: 5'- ATTGAAGTCGACTCACTTCATCCAAGGGCCTAG-3'
E1A-12S	pLPC 12S E1A (Addgene #18740)	F: 5'-AACACAGGTGTCGTGACGCGATGAGACATATTATCTGCCACGG-3'<br>R: 5'-GCTAGCTACTAGCTAGTCGATTATGGCCTGGGGCGTTTAC-3'
ERAS	FUW-TetO-lox-ERAS (Addgene #52417)	F: 5'- AACACAGGTGTCGTGACGCGATGGAGCTGCCAACAAAGC -3'<br>R: 5'-GCTAGCTACTAGCTAGTCGATCAGGCCACAGAGCAGCC-3'
ESRRB	pPB-PGK-hESRRB (Addgene #60434)	F: 5'- AACACAGGTGTCGTGACGCGATGTCCTCGGACGACAGG -3'<br>R: 5'- GCTAGCTACTAGCTAGTCGAATTACATGGTGAGCCAGAGATG-3'
ESRRG	RT-PCR from human PSCs	F: 5'- AACACAGGTGTCGTGACGCGATGGATTCGGTAGAACTTTGC -3'<br>R: 5'- GCTAGCTACTAGCTAGTCGATCAGACCTTGGCCTCCAAC -3'
GASC1	RT-PCR from human PSCs	F: 5'- AACACAGGTGTCGTGACGCGATGGAGGTGGCCGAGGTG -3'<br>R: 5'- GCTAGCTACTAGCTAGTCGACTAGATTCCCAGCCTTCCCAATTTT AAC -3'
GBX2	RT-PCR from human PSCs	F: 5'- AACACAGGTGTCGTGACGCGATGAGCGCAGCGTTCCCG -3'<br>R: 5'- GCTAGCTACTAGCTAGTCGATCAGGGCCGGGCCTGTTC -3'
GFI1	pENTR-GFI1 (Addgene #16168)	F: 5'- AACACAGGTGTCGTGACGCGATGCCGCGCTCATTTCTCGTCAAA AG -3'<br>R: 5'- GCTAGCTACTAGCTAGTCGATCATTTGAGCCCATGCTGCGTC -3'
KAT2B	KAT2B (Addgene #39002)	F: 5'- AACACAGGTGTCGTGACGCGATGCACCATCATCATCATC -3'<br>R: 5'- GCTAGCTACTAGCTAGTCGATTCGGATCCGTATCCACC-3'
KDM4D	RT-PCR from human PSCs	F: 5'- AACACAGGTGTCGTGACGCGATGGAAACTATGAAGTCTAAGGCC AACTG -3'<br>R: 5'- GCTAGCTACTAGCTAGTCGATTAGGGCACAGGGGCCCA -3'
KHDC1	RT-PCR from human PSCs	F: 5'- AACACAGGTGTCGTGACGCGATGCTGTCGGCCTTCCAG -3'<br>R: 5'- GCTAGCTACTAGCTAGTCGATTACGGATACAGTGAACTCAAATGG -3'
KLF2	pGG137mN1-KLF2-2A-KLF4 (Tapponnier et al., 2017)	F: 5'- GCTACCGGTCGCCACCATGGCGCTGAGTG-3'<br>R: 5'- ATTGAACTCGAGCTACATGTGCCGTTTCATGTGCAG-3'
KLF4	PMXs-hKLF4 (Takahashi et al., 2007)	F: 5'- AACACAGGTGTCGTGACGCGATGGCTGTCAGTGACGCG -3'<br>R: 5'- GCTAGCTACTAGCTAGTCGACTACATGTGCCGTTTCATGTGC.-3'
KLF5	pcDNA3-KLF5 (Addgene #40900)	F: 5'- AACACAGGTGTCGTGACGCGATGGAGAAGTATCTGACACC -3'<br>R: 5'- GCTAGCTACTAGCTAGTCGATCAGTTCTGGTGCCTCTTC-3'

KLF12	FLG-KLF12/ pcDNA3.1-Neo (Addgene #81069)	F: 5'- AACACAGGTGTCGTGACGCGATGAATATCCATATGAAGAGAAAAA CAATAAAG -3'<br>R: 5'- GCTAGCTACTAGCTAGTCGATCACACCAACATATGCCTC -3'
KLF17	cDNA synthesis	Cloning between AfeI and XhoI sites in pW10
Large T	pSG5 Large T (Addgene #9053)	F: 5'- AACACAGGTGTCGTGACGCGATGGATAAAGTTTTAAACAGAGAG -3'<br>R: 5'- GCTAGCTACTAGCTAGTCGATTATGTTTCAGGTTCAGG -3'
MYC	PMXs-hc-MYC (Takahashi et al., 2007)	F: 5'-AACACAGGTGTCGTGACGCGATGCCCCTCAACGTTAGC -3'<br>R: 5'- GCTAGCTACTAGCTAGTCGATCACGCACAAGAGTTCCG -3'
NR5A2	RT-PCR from human PSCs	F: 5'- AACACAGGTGTCGTGACGCGATGTCTTCTAATTCAGATACTG -3'<br>R: 5'- GCTAGCTACTAGCTAGTCGATTATGCTCTTTTGGCATG -3'
OOEP	cDNA synthesis	Cloning between AfeI and XhoI sites in pW10
PRMT6	GST-PRMT6 (Addgene #34700)	F: 5'- AACACAGGTGTCGTGACGCGATGTCGCAGCCCAAGAAAAG -3'<br>R: 5'- GCTAGCTACTAGCTAGTCGATCAGTCCTCCATGGCAAAG-3'
SALL4	RT-PCR from human PSCs	F: 5'- AACACAGGTGTCGTGACGCGATGTCGAGGCGCAAGCAG -3'<br>R: 5'- GCTAGCTACTAGCTAGTCGATTAGCTGACCGCAATCTTGTTTTC- 3'
SMAD7	pCMV5-SMAD7-HA (Addgene #11733)	F: 5'- GCTACCGGTCGCCACCATGTTCAGGACCAAACG-3'<br>R: 5'- ATTGAACTCGAGCTACCGGCTGTTGAAGATGAC-3'
SOX2	PMXs-hSOX2 (Takahashi et al., 2007)	F: 5'- AACACAGGTGTCGTGACGCGATGTACAACATGATGGAGACG -3'<br>R: 5'- GCTAGCTACTAGCTAGTCGATCACATGTGTGAGAGGGG -3'
SUV39H1	RT-PCR from human PSCs	F: 5'- AACACAGGTGTCGTGACGCGATGGTGGGGATGAGTCGC -3'<br>R: 5'- GCTAGCTACTAGCTAGTCGACTAGAAGAGGTATTTGCGGC -3'
TBX3	cDNA synthesis	Cloning between AfeI and XhoI sites in pW10
TFAP2C	tetON-TFAP2C-2A-RFP	F 5'-GCTACCGGTCGCCACCATGTTGTGGAAAATAACCGATAATG-3'<br>R 5'-ATTGAACTCGAGTTATTTCCTGTGTTTCTCCATTTTCTC-3'
TFCP2L1	RT-PCR from human PSCs	F: 5'- AACACAGGTGTCGTGACGCGATGCTCTTCTGGCACACG -3'<br>R: 5'- GCTAGCTACTAGCTAGTCGATCAGAGTCCACATTTCAGG -3'
TRIM8	RT-PCR from human PSCs	F: 5'- GCTACCGGTCGCCACCATGGCGGAGAATTGGAAG-3'<br>R: 5'- ATTGAAGTCGACTTAGCTCGTCACGTAGTGTTTG-3'
USP21	Flag-HA-USP21 (Addgene #22574)	F: 5'- GCTACCGGTCGCCACCATGCCCCAGGCCTCTGAG-3'<br>R: 5'- ATTGAACTCGAGTCACAGGCACCGGGGTG-3'

Coronado, D., Godet, M., Bourillot, P.Y., Tapponnier, Y., Bernat, A., Petit, M., Afanassieff, M., Markossian, S., Malashicheva, A., Iacone, R*., et al.* (2013). A short G1 phase is an intrinsic determinant of naive embryonic stem cell pluripotency. Stem Cell Res *10*, 118-131.

Tapponnier, Y., Afanassieff, M., Aksoy, I., Aubry, M., Moulin, A., Medjani, L., Bouchereau, W., Mayere, C., Osteil, P., Nurse-Francis, J*., et al.* (2017). Reprogramming of rabbit induced pluripotent stem cells toward epiblast and chimeric competency using Kruppel-like factors. Stem Cell Res *24*, 106-117.

Negre, D., Mangeot, P.E., Duisit, G., Blanchard, S., Vidalain, P.O., Leissner, P., Winter, A.J., Rabourdin-Combe, C., Mehtali, M., Moullier, P., et al. (2000). Characterization of novel safe lentiviral vectors derived from simian immunodeficiency virus (SIVmac251) that efficiently transduce mature human dendritic cells. Gene Ther *7*, 1613-1623.

Supplementary Table 4: List of primers used for genomic PCR amplification of proviral DNAs and size of amplified DNA fragments

cDNA	Size (nt)	Primers
BMI1	542	Forward 5'-CCAAGTTCACAAGACCAGAC-3'<br>Reverse 5'-GGTGACTGATCTTCATTCTT-3'
CCNE1	748	Forward 5'-CCTCGGATTATTGCACCATC-3'<br>Reverse 5'-TCCCCGCTCCCTTATAACC-3'
DAX1	856	Forward 5'-GGGTAAAGAGGCGCTACCA-3'<br>Reverse 5'-ACAGAGCATTTCCAGCATCA-3'
DPPA2	737	Forward 5'-GAATTTCTTGGAGGGGGAAG-3'<br>Reverse 5'-TCCTCCTGTGAGTGGTAGGC-3'
DPPA4	709	Forward 5'-GGACCTCCACAGAGAAGTCG-3'<br>Reverse 5'-AAACTGCAGGTGAACCCAAC-3'
DPPA5	311	Forward 5'-TGAAAGTTCCCGAAGACCTG-3'<br>Reverse 5'-TCACTTCATCCAAGGGCCTA-3'
E1A-12S	515	Forward 5'-CCACCTCCTAGCCATTTTGA-3'<br>Reverse 5'-ACTGGTTTAATGGGGCACAG-3'
ERAS	781	Forward 5'-AACCGGTGCCTAGAGAAGGT-3'<br>Reverse 5'-CTCCTGGACCCTCTGGATCT-3'
ESRRB	927	Forward 5'-GAGAGCAGCCCATACCTGAG-3'<br>Reverse 5'-GTGAGCCAGAGATGCTTTCC-3'
ESRRG	844	Forward 5'-AGATCCCCAGACCAAGTGTG-3'<br>Reverse 5'-AAGCTTCTGAACGGCTTCAA-3'
GASC1	969	Forward 5'-CATGTGGAAGACCACGTTTG-3'<br>Reverse 5'-TCGGATGGGTCTGATTTCTC-3'
GBX2	731	Forward 5'-GCTCACCTCTACGCTCATGG-3'<br>Reverse 5'-GCTGCTGATGCTGACTTCTG-3'
GFI1	975	Forward 5'-CGGAGTTTGAGGACTTCTGG-3'<br>Reverse 5'-CCTTCCTCTGGAAACCCTTC-3'
KAT2B	387	Forward 5'-CCAATCCATGGGAAAAGAGA-3'<br>Reverse 5'-CGGATCCGTATCCACCTTTA-3'
KDM4D	1221	Forward 5'-GTGCCCAGAATCCAAATTGT-3'<br>Reverse 5'-TGCGTAGCAGTGCCACTAAC-3'
KHDC1	900	Forward 5'-AACCGGTGCCTAGAGAAGGT-3'<br>Reverse 5'-AGGAACGCTAAGTCACACGG-3'
KLF2	699	Forward 5'-TGCGTTCTATTACCCCGAAC-3'<br>Reverse 5'-CAGTGGTAGGGCTTCTCACC-3'
KLF4	1145	Forward 5'-AACCGGTGCCTAGAGAAGGT-3'<br>Reverse 5'-GTGCCTTGAGATGGGAACTC-3'
KLF5	924	Forward 5'-AACCGGTGCCTAGAGAAGGT-3'<br>Reverse 5'-AGTTAACTGGCAGGGTGGTG-3'
KLF12	728	Forward 5'-TCAGCGTCATCTTCGTCAAC-3'<br>Reverse 5'-AATGGCTTCACTCCCGTATG-3'
KLF17	743	Forward 5'-TCTGGAGTGCACACCTCTTG-3'<br>Reverse 5'-TGGGAGCGTTTGGTATAAGC-3'
Large T	1147	Forward 5'-CTGACTTTGGAGGCTTCTGG-3'<br>Reverse 5'-GCTCAAAGTTCAGCCTGTCC-3'
MYC	1236	Forward 5'-AACCGGTGCCTAGAGAAGGT-3'<br>Reverse 5'-TTGTGTGTTCGCCTCTTGAC-3'
NANOG	673	Forward 5'-GATTTGTGGGCCTGAAGAAA-3'<br>Reverse 5'-GAATTTGGCTGGAACTGCAT-3'
NR5A2	874	Forward 5'-AGCTATGCCCTCTGACCTGA-3'<br>Reverse 5'-CTGCTGCGGGTAGTTACACA-3'
OOEP	412	Forward 5'-ATGGTCGATGATGCTGGTG-3'<br>Reverse 5'-CATGGGCCTTCAAGTTCTTC-3'
PRMT6	873	Forward 5'-AACCGGTGCCTAGAGAAGGT-3'<br>Reverse 5'-AATCCCTGCACAACGATCTC-3'
SALL4	1229	Forward 5'-GAAACCCCAGCACATCAACT-3'<br>Reverse 5'-TGCTTAACAAAGGGGTCATCC-3'
SMAD7	270	Forward 5'-AACCGGTGCCTAGAGAAGGT-3'

		Reverse 5’-CTCCTCCTCCACCTCCCC-3’
SOX2	883	Forward 5’-AACCGGTGCCTAGAGAAGGT-3’<br>Reverse 5’-GACTTGACCACCGAACCCAT-3’
SUV39H1	927	Forward 5’-GCGTATCCTCAAGCAGTTCC-3’<br>Reverse 5’-CAATACGGACCCGCTTCTTA-3’
TBX3	790	Forward 5’-GTCATTCCTGGGACAAGCAT-3’<br>Reverse 5’-GAATTCAGTTTCGGGGAACA-3’
TFAP2C	1156	Forward 5’-CACTGGAGTCGCCGAATATC-3’<br>Reverse 5’-CTGGTCTCCAGGGTTCATGT-3’
TFCP2L1	791	Forward 5’-ACGAGAATGGGGAGTACACG-3’<br>Reverse 5’-TGTGCTGAGGACAAAACAGG-3’
TRIM8	730	Forward 5’-AACCGGTGCCTAGAGAAGGT-3’<br>Reverse 5’-CATGAGCATCTTCCGGAT-3’
USP21	793	Forward 5’-AACCGGTGCCTAGAGAAGGT-3’<br>Reverse 5’-GCCCAGAAGGAGTGTGTGAT-3’
TBP	550	Forward 5’-*CTCTGGCAGGTTCAGTTTCC*-3’<br>Reverse 5’-ACAACTGAGCCACAATGCTG-3’

**GFP**	95	Forward 5’-GGCACAAGCTGGAGTACAAC-3’<br>Reverse 5’-GTGGCGGATCTTGAAGTTCAC-3’
**hKLF2:V5**	1050	Forward 5’- CGTGCTGGACTTCATCCTGTC-3’<br>Reverse 5’- GGATAGGCTTACCCATGTGCC-3’
**HA:ERAS**	597	Forward 5’ -CCAGATTACGCTATGGAGCT- 3’<br>Reverse 5’- GCATCTCCAGCAGTGGTCA- 3’
**FLAG:PRMT6**	830	Forward 5’- CCAGATTACGCTATGGAGCT- 3’<br>Reverse 5’- GCATCTCCAGCAGTGGTCA- 3’
**Integration site at chromosome 1**	1720	Forward 5’-GGCACAAGCTGGAGTACAAC-3’<br>Reverse 5’ -TGGGTGGCTTCTGTCGATTT- 3’

Uncropped Western blots

Uncropped Western blot images from Figure S3 showing the analysis of AKT and phosphorylated AKT (Ph-AKT) expression in B19_KF and KEP#37_KF cells

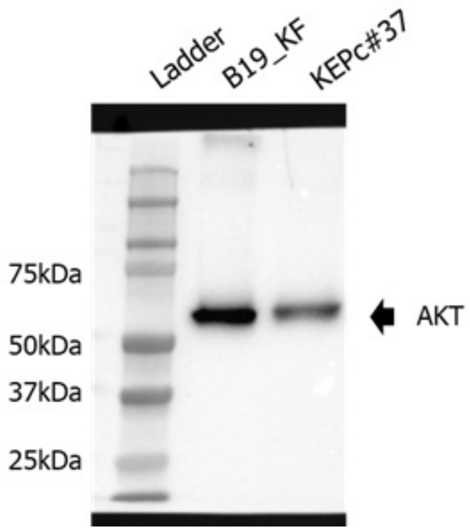

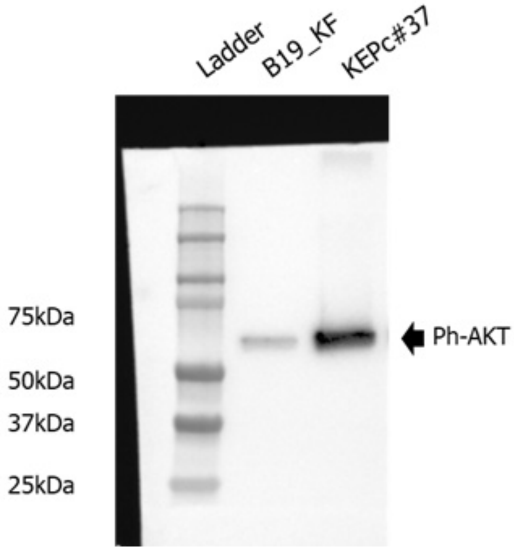

Uncrppoed Western blot images from Figure S6 showing the analysis of AKT and phosphorylated AKT (Ph-AKT) in B19_VAL, KEPc#13_VAL+S, KEPc#13_VAL+S-S, KEPc#18_VAL+S, and KEPc#18_VAL+S-S cells (ACTIN is used as a loading control).

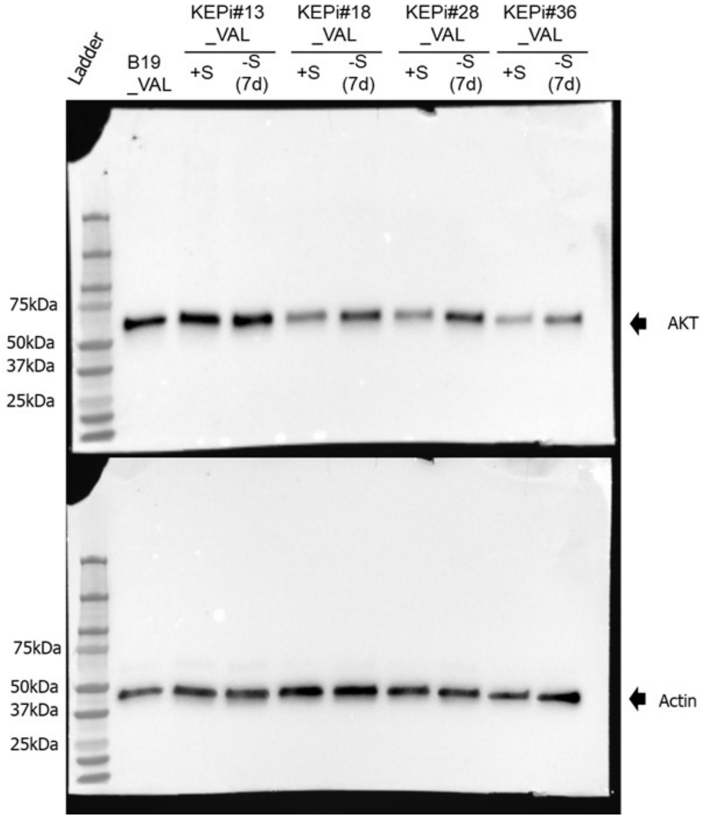

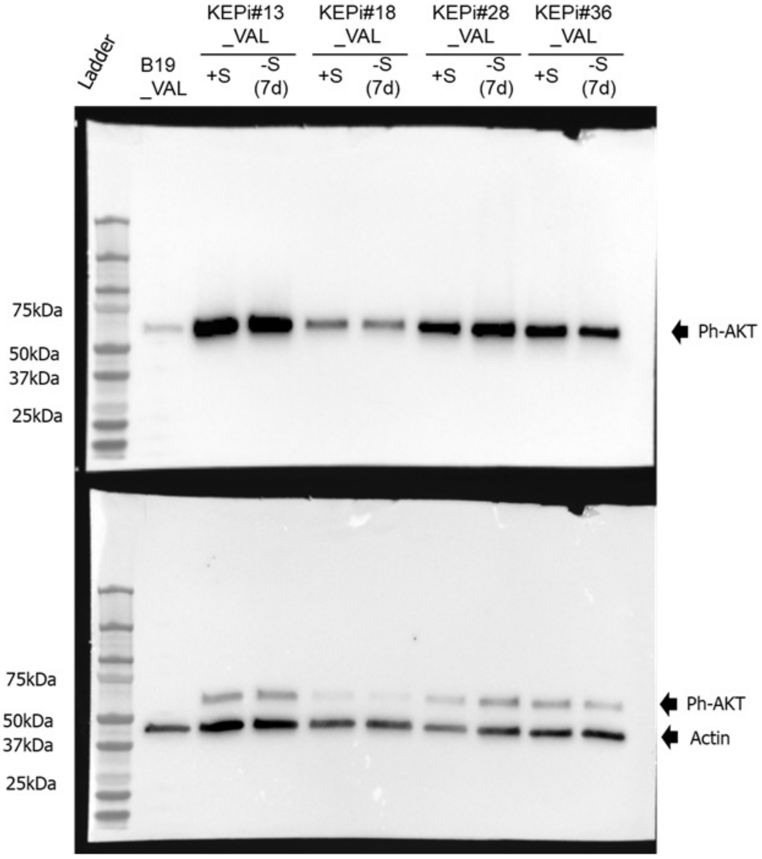

Supplement: Supplementary file 1 — Supplementary Information [file 41467_2025_60314_MOESM1_ESM.pdf]
