# Supplementary material for: Efficient generation of germline chimeras in a non-rodent species using rabbit induced pluripotent stem cells
Source: Nat Commun. 2025 Jun 4;16:5165. doi: 10.1038/s41467-025-60314-2 (PMC12134177; doi:10.1038/s41467-025-60314-2)
Supplement: Supplementary file 2 — Reporting Summary [file 41467_2025_60314_MOESM2_ESM.pdf]

nature portfolio

Corresponding author(s): Pierre SAVATIER

Last updated by author(s): Dec 1, 2024

Reporting Summary

Nature Portfolio wishes to improve the reproducibility of the work that we publish. This form provides structure for consistency and transparency in reporting. For further information on Nature Portfolio policies, see our Editorial Policies and the Editorial Policy Checklist.

Statistics

For all statistical analyses, confirm that the following items are present in the figure legend, table legend, main text, or Methods section.

n/a	Confirmed	
☐	☒	The exact sample size (*n*) for each experimental group/condition, given as a discrete number and unit of measurement
☐	☒	A statement on whether measurements were taken from distinct samples or whether the same sample was measured repeatedly
☐	☒	The statistical test(s) used AND whether they are one- or two-sided<br>*Only common tests should be described solely by name; describe more complex techniques in the Methods section.*
☒	☐	A description of all covariates tested
☐	☒	A description of any assumptions or corrections, such as tests of normality and adjustment for multiple comparisons
☒	☐	A full description of the statistical parameters including central tendency (e.g. means) or other basic estimates (e.g. regression coefficient) AND variation (e.g. standard deviation) or associated estimates of uncertainty (e.g. confidence intervals)
☐	☒	For null hypothesis testing, the test statistic (e.g. *F*, *t*, *r*) with confidence intervals, effect sizes, degrees of freedom and *P* value noted<br>*Give P values as exact values whenever suitable.*
☒	☐	For Bayesian analysis, information on the choice of priors and Markov chain Monte Carlo settings
☒	☐	For hierarchical and complex designs, identification of the appropriate level for tests and full reporting of outcomes
☒	☐	Estimates of effect sizes (e.g. Cohen's *d*, Pearson's *r*), indicating how they were calculated

Our web collection on statistics for biologists contains articles on many of the points above.

Software and code

Policy information about availability of computer code

Data collection	Images of cells and embryos were acquired on various microscopes equipped with commercial softwares:<br>1) Nikon conventional fluorescence microscope equipped with NIS-Elements imaging software v4.30.<br>2) Leica confocal equipped with LAS software AF version.<br>For qPCR and RT-qPCR, data acquisition were performed with StepOne software v.2.3.<br>Flow cytometry sorting was performed with BD FACS Diva software.<br>Western Blots imaging was performed with ImageLab imaging software v5.1
Data analysis	Image analysis and quantification was performed with Open source software FIJI and ChromosomeJ plugin for karyotyping.<br>qPCR and RT-qPCR, data acquisition, and analysis were performed with StepOne software v.2.3.<br>Flow cytometry analysis was performed using BD FACSDiva software.<br>Bioinformatics Analysis : All analyses were executed using R software (version 4.1.2), and several related packages such as FeatureCounts (version 1.5.0-p2), DESeq2 R package (version 1.34.0), pheatmap R package (version 1.9.12), VennDiagram package (version 1.7.3), ggplot2 package (version 3.4.1) and Seurat R package (version 4.3.0). EnrichR web tool was used for KEGG pathway enrichment analysis.<br>Statistical analysis: R base package (version 4.1.2) and SuperPlotsOfData web tools

For manuscripts utilizing custom algorithms or software that are central to the research but not yet described in published literature, software must be made available to editors and reviewers. We strongly encourage code deposition in a community repository (e.g. GitHub). See the Nature Portfolio guidelines for submitting code & software for further information.

Data

Policy information about availability of data

All manuscripts must include a data availability statement. This statement should provide the following information, where applicable:

- Accession codes, unique identifiers, or web links for publicly available datasets
- A description of any restrictions on data availability
- For clinical datasets or third party data, please ensure that the statement adheres to our policy

RNA-seq data have been deposited at GEO (GSE250288).
https://www.ncbi.nlm.nih.gov/geo/query/acc.cgi?acc=GSE250288
Original western blot images have been included in the Source Data files.
Confocal microscopy data reported in this paper will be shared by the lead contact upon request.

Research involving human participants, their data, or biological material

Policy information about studies with human participants or human data. See also policy information about sex, gender (identity/presentation), and sexual orientation and race, ethnicity and racism.

Reporting on sex and gender	*Use the terms sex (biological attribute) and gender (shaped by social and cultural circumstances) carefully in order to avoid confusing both terms. Indicate if findings apply to only one sex or gender; describe whether sex and gender were considered in study design; whether sex and/or gender was determined based on self-reporting or assigned and methods used. Provide in the source data disaggregated sex and gender data, where this information has been collected, and if consent has been obtained for sharing of individual-level data; provide overall numbers in this Reporting Summary. Please state if this information has not been collected. Report sex- and gender-based analyses where performed, justify reasons for lack of sex- and gender-based analysis.*
Reporting on race, ethnicity, or other socially relevant groupings	*Please specify the socially constructed or socially relevant categorization variable(s) used in your manuscript and explain why they were used. Please note that such variables should not be used as proxies for other socially constructed/relevant variables (for example, race or ethnicity should not be used as a proxy for socioeconomic status). Provide clear definitions of the relevant terms used, how they were provided (by the participants/respondents, the researchers, or third parties), and the method(s) used to classify people into the different categories (e.g. self-report, census or administrative data, social media data, etc.) Please provide details about how you controlled for confounding variables in your analyses.*
Population characteristics	*Describe the covariate-relevant population characteristics of the human research participants (e.g. age, genotypic information, past and current diagnosis and treatment categories). If you filled out the behavioural & social sciences study design questions and have nothing to add here, write "See above."*
Recruitment	*Describe how participants were recruited. Outline any potential self-selection bias or other biases that may be present and how these are likely to impact results.*
Ethics oversight	*Identify the organization(s) that approved the study protocol.*

Note that full information on the approval of the study protocol must also be provided in the manuscript.

Field-specific reporting

Please select the one below that is the best fit for your research. If you are not sure, read the appropriate sections before making your selection.

☒ Life sciences ☐ Behavioural & social sciences ☐ Ecological, evolutionary & environmental sciences

For a reference copy of the document with all sections, see nature.com/documents/nr-reporting-summary-flat.pdf

Life sciences study design

All studies must disclose on these points even when the disclosure is negative.

Sample size	This question applies to :<br>- RNA sequencing: each experimental condition was analyzed in three independent replicates, as is standard practice for this type of analysis.<br>- For analysis on embryos, sample size was chosen based on similar studies as standard in the field (range 15-110 embryos per condition). The exact numbers of embryos used are reported in each figure.<br>- Cell injection into embryos: colonization rates were calculated from an average of 63 embryos (ranging from 20 to 108, depending on the experimental condition), as shown in the corresponding figures.<br>- For immunostaining analysis on cells, the number of images used per sample and per condition typically ranged from 9 to 18, with at least 100 cells analysed to ensure statistical significance, comparable to the standard practice microscopy analyses (see Figure legends). No statistical methods were used to predetermine the sample sizes.

Data exclusions	No data were excluded from the analysis
Replication	The ability of rabbit KEP cell lines to achieve naïve pluripotency and colonize embryos was demonstrated using at least two independent clones for each experiment, ensuring robustness. Each type of cell reprogramming and embryo colonization experiment was performed at least three times.
Randomization	This question applies to host embryos used in chimera experiments:<br>- Reprogrammed cells (female B19) were injected into male and female embryos in equal proportions.<br>- On each injection day, donor embryos were collected from at least three females , and all embryos were subsequenytly mixed. Embryos used forinjection were randomly picked from to batch.<br>- When different cell lines had to be tested on the same day, the order of injection of the different lines was randomized
Blinding	This issue applies to cell counting in chimeric embryos for quantification of colonization. Cell counting from confocal images was carried out by two different people to avoid bias. This issue also pertains to microinjections of cells into embryos, which were carried out in a blinded fashion by the same person.

Reporting for specific materials, systems and methods

We require information from authors about some types of materials, experimental systems and methods used in many studies. Here, indicate whether each material, system or method listed is relevant to your study. If you are not sure if a list item applies to your research, read the appropriate section before selecting a response.

Materials & experimental systems

n/a	Involved in the study
☐	☒ Antibodies
☐	☒ Eukaryotic cell lines
☒	☐ Palaeontology and archaeology
☐	☒ Animals and other organisms
☒	☐ Clinical data
☒	☐ Dual use research of concern
☒	☐ Plants

Methods

n/a	Involved in the study
☒	☐ ChIP-seq
☐	☒ Flow cytometry
☒	☐ MRI-based neuroimaging

Antibodies

Antibodies used	The information for all antibodies is provided in the 'Experimental procedures' and listed as below:<br>Primary antibodies for Immunofluorescence analysis of cells and embryos include: anti-SOX2 (Bio-Techne, ref AF2018, dilution 1:100), anti-GFP (Invitrogen, ref A10262, dilution 1:200), anti-OCT4 (StemAB, ref 09-0023, dilution 1:200), anti-DPPA5 (R&D systems, ref AF3125, dilution 1:100), anti-OOEP (Abcam ref 185478, dilution 1:100), anti-ubiquityl-Histone H2A Lys119 (Cell Signaling, ref #8240, dilution 1:300), anti-Tri-methyl Histone H3 Lys27 (Cell Signaling, ref #9733, dilution 1:400), anti-Tri-methyl Histone H3 Lys4 (Cell Signaling, ref #9751, dilution 1:400), anti-Histone H3 (acetyl K14) (Abcam, ref ab52946, dilution 1:400), anti-Histone H3 tri methyl K9 (Abcam, ref ab8898, dilution 1:400), anti-Histone H3 (asymmetric di methyl R2) (Abcam, ref ab175007, dilution 1:400), anti-5-methylcytosine (EMD Millipore, ref MABE146, dilution 1:400), anti-CD75 (Abcam, ref ab 77676, dilution 1:1000), anti-TUJ1 (Sigma, ref T8660, 1:10000), anti-smooth muscle actin (SMA; Millipore, ref CBL171, 1:500), anti-LAMININ (Sigma, ref I9393, 1:100), anti-PAX3 (DSHB, ref 3929, 1:500), anti-CD31/PECAM-1 (Bio-techne, ref AF806, 1:100), anti-DESMIN (Abcam, ab32362, 1:200), and anti-SOX17 (Bio-Techne, ref AF1924, 1:25). Secondary antibodies (diluted 1:500 for all of them) include: donkey anti-goat (Alexa Fluor 555) (Invitrogen ref A21432), donkey anti-chicken (Alexa Fluor 488) (Jakson ImmunoResearch, ref 703-545-155), donkey anti-mouse (Alexa Fluor 647, Invitrogen, ref A21448), donkey anti-rabbit (Alexa Fluor 555, Invitrogen, ref A31572) and donkey anti-rabbit (Alexa Fluor 647; Invitrogen, ref A31573), Alexa Fluor 488 goat anti-chicken (Abcam, ab150169), Alexa Fluor 647 donkey anti-mouse IgG (Invitrogen, ref A21448), Alexa Fluor 647 donkey anti-goat IgG (Invitrogen, A21447).<br>Primary antibodies for Western Blotting include: anti-V5 Tag (Invitrogen, ref R96025, 1:500, anti-HA (Sigma-Aldrich, ref H6908, 1:500), anti-Akt (pan) (Cell Signaling, ref #4691, 1:1000), anti-Phospho-Akt (Ser473) (Cell Signaling, ref #4060, 1:1000), and anti-beta-actin (Sigma-Aldrich, ref A3854, 1:10000) and HRP-conjugated secondary antibody (Jackon ImmunoResearch anti-rabbit ref 211-032-171 and anti-mouse ref 115-035-146, dilution 1:5000).<br>CD75 antibody (Abcam, ref ab77676; dilution 1:1000) and Alexa Fluor™ Plus 647 (Invitrogen, ref A32728; dilution 1:200) were used as primary and secondary antibody for Flow cytometry analysis and sorting.
Validation	All antibodies used in this study are commercially available and validated in several species as indicated on manufacturer's websites (listed below). These antibodies have been validated in other publications (Osteil, P. et al. 2013; Osteil, P. et al. 2015; Tapponnier, Y. et al. 2017; Bouchereau, W. et al. 2022). The staining we obtained corresponds to expected signals (data provided in the manuscript (Figure 1-7) and Supplementary data).<br>1. anti-SOX2 (Bio-Techne, Goat, catalog # AF2018, dilution 1:100): https://www.bio-techne.com/p/antibodies/human-mouse-rat-sox2-antibody_af2018#product-documents<br>2. anti-GFP (Invitrogen, Chicken, catalog #A10262, dilution 1:200): https://www.thermofisher.com/antibody/product/GFP-Antibody-Polyclonal/A10262<br>3. anti-OCT4 (StemAB, rabbit, catalog # 09-0023, dilution 1:200): https://www.reprocell.com/product-catalog/antibodies-and-staining-kits/stemab-oct4-antibody-affinity-purified-rabbit-anti-mouse-human

4. anti-DPPA5 (R&D systems, goat, catalog # AF3125, dilution 1:100): https://www.rndsystems.com/products/human-dppa5-esg1-antibody_af3125
5. anti-OOEP (Abcam, rabbit, catalog #ab185478, dilution 1:100): https://doc.abcam.com/datasheets/inactive/ab185478/en-us/ooep-antibody-ab185478.pdf
6. anti-ubiquityl-Histone H2A Lys119 (Cell Signaling, rabbit, catalog #8240, dilution 1:300): https://www.cellsignal.com/products/primary-antibodies/ubiquityl-histone-h2a-lys119-d27c4-xp-rabbit-mab/8240?srsltid=AfmBOoqKwidmkjrUsAQSwbij1g_JcqLnWdzM3oyCz57jOpvSiqyrr8yN
7. anti-Tri-methyl Histone H3 Lys27 (Cell Signaling, rabbit, catalog #9733, dilution 1:400): https://www.cellsignal.com/products/primary-antibodies/tri-methyl-histone-h3-lys27-c36b11-rabbit-mab/9733?srsltid=AfmBOoqljnqdRRfxvGb018D8ziS45Fixnjw55QEFmevgV91M1DCVIfQh
8. anti-Tri-methyl Histone H3 Lys4 (Cell Signaling, rabbit, catalog # #9751, dilution 1:400): https://www.cellsignal.com/products/primary-antibodies/tri-methyl-histone-h3-lys4-c42d8-rabbit-mab/9751?srsltid=AfmBOorKGwrXV0OHv_VERrkTl9GXLmMt_72stVyNQos9M6HpnF9DCJ9g
9. anti-Histone H3 (acetyl K14) (Abcam, rabbit, catalog # ab52946, dilution 1:400): https://www.abcam.com/en-us/products/primary-antibodies/histone-h3-acetyl-k14-antibody-ep964y-chip-grade-ab52946?srsltid=AfmBOoq2HUxnfKk87954z1UeXwO7D15UtH2Z41pxLPA9bnemZIqtT2nl
10. anti-Histone H3 tri methyl K9 (Abcam, rabbit, catalog # ab8898, dilution 1:400): https://www.abcam.com/en-us/products/primary-antibodies/histone-h3-tri-methyl-k9-antibody-chip-grade-ab8898?srsltid=AfmBOoo-HbJkmbb04GZJ_zHqOjaac9qyH8g4DKmhB1hbEvKmcsd2LBNJ
11. anti-Histone H3 (asymmetric di methyl R2) (Abcam, rabbit, catalog # ab175007, dilution 1:400): https://www.citeab.com/antibodies/2436223-ab175007-anti-histone-h3-asymmetric-di-methyl-r2-a
12. anti-5-methylcytosine (EMD Millipore, mouse, catalog # MABE146, dilution 1:400): https://www.merckmillipore.com/FR/fr/product/Anti-5-methylcytosine-Antibody-clone-33D3,MM_NF-MABE146?ReferrerURL=https%3A%2F%2Fwww.google.com%2F
13. anti-CD75 (Abcam, mouse, catalog # ab 77676, dilution 1:1000): https://doc.abcam.com/legacy-unpublished/datasheets/com/datasheet_77676.pdf?_gl=1*cha3mi*_gcl_au*MTE4MjAzMTk3OS4xNzMxNTc5NzA1*_ga*MjA4NzQ4MTYzMC4xNzMxNTc5NTUx*_ga_LTF3E70SKQ*MTczMzI2NDc0OC43LjEuMTczMzI2NTIxNi4zNy4wLjA.
14. anti-TUJ1 (Sigma, mouse, catalog # T8660, 1:10000) : https://www.citeab.com/antibodies/2286821-t8660-monoclonal-anti-tubulin-iii-antibody-produce
15. anti-smooth muscle actin (SMA; mouse, Millipore, catalog # CBL171, 1:500): https://www.merckmillipore.com/FR/fr/product/Anti-Actin-Antibody-smooth-muscle-clone-ASM-1,MM_NF-CBL171?ReferrerURL=https%3A%2F%2Fwww.google.com%2F
16. anti-LAMININ (Sigma, rabbit, catalog # l9393, 1:100) : https://www.sigmaaldrich.com/FR/fr/product/sigma/l9393?srsltid=AfmBOoqhrmb09H6yLlkUEt-J_xiwLdDFjWrnIDxQbzOj1M8Meea0Xb5Q
17. anti-PAX3 (DSHB, mouse, catalog # 3929, 1:500) : https://dshb.biology.uiowa.edu/Pax3?quantity=1&product-form=1
18. anti-CD31/PECAM-1 (Bio-techne, sheep, catalog # AF806, 1:100): https://www.rndsystems.com/products/human-cd31-pecam-1-antibody_af806
19. anti-DESMIN (Abcam, rabbit, ab32362, 1:200): https://www.abcam.com/en-us/products/primary-antibodies/desmin-antibody-y66-cytoskeleton-marker-ab32362?srsltid=AfmBOoophjDdeWmXfKmog28XfwvI4zjlTFmDf5CoBB6IDtTVJnMfYmuz
20. anti-SOX17 (Bio-Techne, goat, catalog # AF1924, 1:25) : https://www.rndsystems.com/products/human-sox17-antibody_af1924?gad_source=1&gclid=CjwKCAiA9bq6BhAKEiwAH6bqoHkmdTktidGGv2ZytU1QKZsP7L1PjBvkotViHVeggLZE08JoLZXqfxoCJAUQAvD_BwE&gclsrc=aw.ds
21. donkey anti-goat (Alexa Fluor 555) (Invitrogen catalog # A21432): https://www.thermofisher.com/antibody/product/Donkey-anti-Goat-IgG-H-L-Cross-Adsorbed-Secondary-Antibody-Polyclonal/A-21432
22. donkey anti-chicken (Alexa Fluor 488) (Jakson ImmunoResearch, catalog # 703-545-155): https://www.jacksonimmuno.com/catalog/products/703-545-155
23. donkey anti-mouse (Alexa Fluor 647, Invitrogen, catalog # A21448): https://www.thermofisher.com/antibody/product/Donkey-anti-Sheep-IgG-H-L-Cross-Adsorbed-Secondary-Antibody-Polyclonal/A-21448
24. donkey anti-rabbit (Alexa Fluor 555, Invitrogen, catalog # A31572): https://www.thermofisher.com/antibody/product/Donkey-anti-Rabbit-IgG-H-L-Highly-Cross-Adsorbed-Secondary-Antibody-Polyclonal/A-31572
25. donkey anti-rabbit (Alexa Fluor 647; Invitrogen, catalog # A31573): https://www.thermofisher.com/antibody/product/Donkey-anti-Rabbit-IgG-H-L-Highly-Cross-Adsorbed-Secondary-Antibody-Polyclonal/A-31573
26. Alexa Fluor 488 goat anti-chicken (Abcam, catalog #ab150169): https://www.abcam.com/en-us/products/secondary-antibodies/goat-chicken-igy-h-l-alexa-fluor-488-ab150169?srsltid=AfmBOorIBioM2A7oSXcEZ5DUBl8XmgA4XQU1KcK4BrW-gaw93R3LfnlJ
27. Alexa Fluor 647 donkey anti-mouse IgG (Invitrogen, catalog # A21448): https://www.thermofisher.com/antibody/product/Donkey-anti-Sheep-IgG-H-L-Cross-Adsorbed-Secondary-Antibody-Polyclonal/A-21448
28. Alexa Fluor 647 donkey anti-goat IgG (Invitrogen, catalog # A21447): https://www.thermofisher.com/antibody/product/Donkey-anti-Goat-IgG-H-L-Cross-Adsorbed-Secondary-Antibody-Polyclonal/A-21447
29. anti-V5 Tag (Invitrogen, mouse, catalog # R96025, 1:500); https://www.thermofisher.com/antibody/product/V5-Tag-Antibody-clone-SV5-Pk1-Monoclonal/R960-25
30. anti-HA (Sigma-Aldrich, rabbit, catalog # H6908, 1:500): https://www.sigmaaldrich.com/FR/fr/product/sigma/h6908?srsltid=AfmBOopzne8JO4z0Bw6witQxyjHyXBPAE3SUaB5rT95oE6dsNxVSsTUp
31. anti-Akt (pan) (Cell Signaling, rabbit, catalog # #4691, 1:1000): https://www.cellsignal.com/products/primary-antibodies/akt-pan-c67e7-rabbit-mab/4691?srsltid=AfmBOopLH76LAu_SfVSqBmk9jDYfpDFNdni8HCBNWLFgcRo2hgQhrl96
32. anti-Phospho-Akt (Ser473) (Cell Signaling, rabbit, catalog # #4060, 1:1000); https://www.cellsignal.com/products/primary-antibodies/phospho-akt-ser473-d9e-xp-rabbit-mab/4060?srsltid=AfmBOorRD95W8kKgS6zsAEg1jAaHfFx19-Asz6HPZqg0DvAeOFCyE-4J
33. anti-beta-actin (Sigma-Aldrich, mouse, catalog # A3854, 1:10000); https://www.sigmaaldrich.com/FR/fr/product/sigma/a3854?srsltid=AfmBOoqObhaeW4JYCgYiU6GABV6uLzthPt5I3lZoJ6v3fmbzwvQTaeEo
34. HRP-conjugated anti-rabbit secondary antibody (Jackon ImmunoResearch, mouse, catalog # 211-032-17, dilution 1:5000): https://www.jacksonimmuno.com/catalog/products/211-032-171
35. HRP-conjugated anti-mouse secondary antibody (Jackon ImmunoResearch, goat, catalog # 115-035-146, dilution 1:5000): https://www.jacksonimmuno.com/catalog/products/115-035-146

Eukaryotic cell lines

Policy information about cell lines and Sex and Gender in Research

Cell line source(s)	All experiments were performed using the rabbit iPSC line 19 previously described in Osteil et al. (2013). Biol Open, 2:613-28. Primary embryonic fibroblasts were produced from mixed E12 male and female mouse embryos.
Authentication	The rabbit iPSC line 19 was produced in our laboratory (Osteil et al., 2013, Biol Open, 2:613-28).
Mycoplasma contamination	The rabbit iPSC line B19 and each batch of primary embryonic fibroblasts were tested negative for mycoplasma (tested every three months).
Commonly misidentified lines (See ICLAC register)	na

Animals and other research organisms

Policy information about studies involving animals; ARRIVE guidelines recommended for reporting animal research, and Sex and Gender in Research

Laboratory animals	All rabbits used in the study were sexually mature New Zealand white rabbits.<br>The protocol for the use of DR4 mice was approved by our research laboratory's animal welfare committee (SBEA), composed of the following seven members : four scientists (Dr C. Dehay, Dr H. Kennedy, Dr B. Pain, and Dr E. Procyk), one veterinarian (Dr M. Dirheimer), two research assistants in charge of the animal facility (P. Giroud and A. Bellemin).<br>Mice are housed in groups of five in ventilated cages with bedding, shelter (igloo, tunnel or arch), gnawing material (cotton balls or wooden sticks), and unlimited access to food and drink. The mice are supervised daily by qualified animal keepers.
Wild animals	na
Reporting on sex	Reprogrammed cells (female iPSC B19) were injected into male and female embryos in equal proportions. We did not attempt to determine the sex of the embryos, either before or after cell injection, as this was not relevant to the study. We therefore do not know whether the sex of the embryo has an impact on colonization and somatic chimerism rates.<br>However, the sex of the embryo does have an impact on germline colonization. We injected female iPS cells, which then colonized the germline of female-only embryos to produce female germline chimeras.
Field-collected samples	na
Ethics oversight	All procedures in rabbits were approved by the French ethics committee CELYNE (approval number APAFIS#6438 and APAFIS#39573)

Note that full information on the approval of the study protocol must also be provided in the manuscript.

Plants

Seed stocks	*Report on the source of all seed stocks or other plant material used. If applicable, state the seed stock centre and catalogue number. If plant specimens were collected from the field, describe the collection location, date and sampling procedures.*
Novel plant genotypes	*Describe the methods by which all novel plant genotypes were produced. This includes those generated by transgenic approaches, gene editing, chemical/radiation-based mutagenesis and hybridization. For transgenic lines, describe the transformation method, the number of independent lines analyzed and the generation upon which experiments were performed. For gene-edited lines, describe the editor used, the endogenous sequence targeted for editing, the targeting guide RNA sequence (if applicable) and how the editor was applied.*
Authentication	*Describe any authentication procedures for each seed stock used or novel genotype generated. Describe any experiments used to assess the effect of a mutation and, where applicable, how potential secondary effects (e.g. second site T-DNA insertions, mosiacism, off-target gene editing) were examined.*

Flow Cytometry

Plots

Confirm that:

- [x] The axis labels state the marker and fluorochrome used (e.g. CD4-FITC).
- [x] The axis scales are clearly visible. Include numbers along axes only for bottom left plot of group (a 'group' is an analysis of identical markers).
- [x] All plots are contour plots with outliers or pseudocolor plots.
- [x] A numerical value for number of cells or percentage (with statistics) is provided.

Methodology

Sample preparation	Sample preparation is described in detailed in the "Experimental procedures". Briefly, cells were dissociated to single cell suspension and stained with CD75 primary antibody (Abcam, ref ab77676; dilution 1:50) and Alexa Fluor™ Plus 647 conjugated-goat anti-mouse IgG secondary antibody (Invitrogen, ref A32728; dilution 1:200).
Instrument	LSRFortessa™ X-20 Cell Analyzer (Beckton Dickinson) and FACSAria™ III Sorter (Beckton Dickinson).
Software	BD FACSDivaTM software.
Cell population abundance	The abundance of CD75high cells were confirmed using FACSAria™ III Sorter after FACS-sorting (Beckton Dickinson) (Figure S8)
Gating strategy	Total cells were gated based on FSC-A and SSC-A. Single cells were selected using FSC-H / FSC-A gating. "No antibody" and "no primary antibody" controls were employed to identify any unspecific labeling.

☒ Tick this box to confirm that a figure exemplifying the gating strategy is provided in the Supplementary Information.
